# Efficient large-scale single-step evaluations and indirect genomic prediction of genotyped selection candidates

**DOI:** 10.1186/s12711-023-00808-z

**Published:** 2023-06-08

**Authors:** Jeremie Vandenplas, Jan ten Napel, Saeid Naderi Darbaghshahi, Ross Evans, Mario P. L. Calus, Roel Veerkamp, Andrew Cromie, Esa A. Mäntysaari, Ismo Strandén

**Affiliations:** 1 Wageningen University and Research, P.O. Box 338, 6700 AH Wageningen, The Netherlands; 2 Irish Cattle Breeding Federation, Highfield House, Newcestown Road, Bandon, Cork Ireland; 3 Natural Resources Institute Finland (Luke), Jokioinen, Finland

## Abstract

**Background:**

Single-step genomic best linear unbiased prediction (ssGBLUP) models allow the combination of genomic, pedigree, and phenotypic data into a single model, which is computationally challenging for large genotyped populations. In practice, genotypes of animals without their own phenotype and progeny, so-called genotyped selection candidates, can become available after genomic breeding values have been estimated by ssGBLUP. In some breeding programmes, genomic estimated breeding values (GEBV) for these animals should be known shortly after obtaining genotype information but recomputing GEBV using the full ssGBLUP takes too much time. In this study, first we compare two equivalent formulations of ssGBLUP models, i.e. one that is based on the Woodbury matrix identity applied to the inverse of the genomic relationship matrix, and one that is based on marker equations. Second, we present computationally-fast approaches to indirectly compute GEBV for genotyped selection candidates, without the need to do the full ssGBLUP evaluation.

**Results:**

The indirect approaches use information from the latest ssGBLUP evaluation and rely on the decomposition of GEBV into its components. The two equivalent ssGBLUP models and indirect approaches were tested on a six-trait calving difficulty model using Irish dairy and beef cattle data that include 2.6 million genotyped animals of which about 500,000 were considered as genotyped selection candidates. When using the same computational approaches, the solving phase of the two equivalent ssGBLUP models showed similar requirements for memory and time per iteration. The computational differences between them were due to the preprocessing phase of the genomic information. Regarding the indirect approaches, compared to GEBV obtained from single-step evaluations including all genotypes, indirect GEBV had correlations higher than 0.99 for all traits while showing little dispersion and level bias.

**Conclusions:**

In conclusion, ssGBLUP predictions for the genotyped selection candidates were accurately approximated using the presented indirect approaches, which are more memory efficient and computationally fast, compared to solving a full ssGBLUP evaluation. Thus, indirect approaches can be used even on a weekly basis to estimate GEBV for newly genotyped animals, while the full single-step evaluation is done only a few times within a year.

**Supplementary Information:**

The online version contains supplementary material available at 10.1186/s12711-023-00808-z.

## Background

The original single-step genomic best linear unbiased prediction (ssGBLUP) genomic evaluation studies [1, 2] presented a theoretically well-justified model for genetic evaluation, which allows the inclusion of pedigree and phenotypes of genotyped and non-genotyped animals. Practical implementation of ssGBLUP has met both computational and modelling challenges (e.g., [3, 4]). Several approaches have been presented to allow efficient modelling and solving of ssGBLUP (e.g., [5–8]). In practice, estimated breeding values for dairy and beef cattle are computed three to four times for a trait or trait group during a year, but genotypes for newly born animals become available almost continuously throughout the year. Because the computation of the full data ssGBLUP predictions is demanding, computationally efficient calculation of breeding values for these newly genotyped animals is desirable. We will use the term genotyped selection candidate for a newly genotyped animal without progeny and own phenotype and for which we would like to compute genomic predictions.

Legarra and Ducrocq [9], Fernando et al. [6], Liu et al. [7], and Taskinen et al. [10] presented single-step approaches, which are hereinafter denoted ssSNPBLUP where the mixed model equations (MME) include single nucleotide polymorphism (SNP) effects. Solutions of the SNP effects allow a simple approach to estimate GEBV for genotyped selection candidates without the need to solve the full updated MME. Lourenco et al. [11] presented a similar approach for ssGBLUP where the SNP effects are estimated using a formula derived from a GBLUP model. Pimentel et al. [12] presented and tested approximate approaches for the prediction of selection candidates in ssGBLUP, which were based on SNP-BLUP or GBLUP and facilitated by including the so-called residual polygenic (RPG) effect. Liu et al. [13] presented formulas for predicting breeding values of genotyped selection candidates when solutions from ssSNPBLUP were available and the model had an RPG effect.

Prediction of breeding values of genotyped selection candidates can be integrated with the existing computational approaches used for solving the full data ssGBLUP. Mäntysaari et al. [8] presented an efficient computational approach with T-factoring named ssGTBLUP for single-step genomic evaluations. This approach assumes that the genomic relationship matrix has the form $\mathbf{G}=\mathbf{Z}\mathbf{Z}^{\mathbf{\prime}}+\mathbf{C}$, where $\mathbf{Z}$ is a centered and scaled genotype marker matrix and $\mathbf{C}$ is a non-singular easily invertible regularization matrix. The main computational step in solving the MME of ssGBLUP by an iterative method is the calculation of ${\mathbf{G}}^{-1}$ times a vector product in each iteration. In ssGTBLUP, this product was shown to require two products involving a rectangular matrix of size $m$ by $n$ where $m$ is the number of SNPs and $n$ is the number of genotyped animals. Consequently, computational work increases linearly with the number of genotyped animals $n$ instead of quadratically as in regular ssGBLUP. According to Mäntysaari et al. [3], absorption of the SNP effects in the MME of the ssSNPBLUP model proposed by Liu et al. [7] leads to ssGTBLUP where $\mathbf{C}$ is the pedigree-based relationship matrix among genotyped animals multiplied by the proportion of RPG effect.

In this study, we present a unified model for ssGTBLUP [8] and ssSNPBLUP [7], which extends the general regularization matrix $\mathbf{C}$ used in ssGTBLUP to be integrated in ssSNPBLUP. Furthermore, we present efficient indirect approaches for both ssGTBLUP and ssSNPBLUP models that allow the prediction of GEBV for the genotyped selection candidates under different regularization matrices. We investigate and compare the performance of the ssGTBLUP, ssSNPBLUP and indirect approaches by using a multi-trait model with more than 2.6 million genotyped animals.

## Methods

### ssGTBLUP and ssSNPBLUP

We investigated two computational approaches for single-step genomic evaluation. First, we use a general notation to describe the ssGTBLUP approach proposed by Mäntysaari et al. [8]. Second, ssGTBLUP is used to derive the ssSNPBLUP approach by Liu et al. [7]. In spite of apparent differences in the MME of these approaches, we show how, in theory, they are computationally similar.

#### The ssGTBLUP approach

A standard univariate mixed model for ssGBLUP can be written as:

1 $$\mathbf{y}=\mathbf{X}\mathbf{b}+\left[\begin{array}{cc}{\mathbf{W}}_{n}& {\mathbf{0}}\\ {\mathbf{0}}& {\mathbf{W}}_{g}\end{array}\right]\left[\begin{array}{c}{\mathbf{u}}_{n}\\ {\mathbf{u}}_{g}\end{array}\right]+\mathbf{e},$$

where $\mathbf{y}$ is the vector of records, $\mathbf{b}$ is the vector of fixed effects, ${\mathbf{u}}_{n}$ is the vector of additive genetic effects for the non-genotyped animals, ${\mathbf{u}}_{g}$ is the vector of additive genetic effects for the genotyped animals, and $\mathbf{e}$ is the vector of residuals. The matrices $\mathbf{X}$, ${\mathbf{W}}_{n}$, and ${\mathbf{W}}_{g}$ relate records in $\mathbf{y}$ to the corresponding effects.

We assume normally distributed additive genetic effects $$\mathbf{u}^{\mathbf{\prime}}=\left[\begin{array}{cc}{\mathbf{u}}_{n}^{\mathbf{\prime}}& {\mathbf{u}}_{g}^{\mathbf{\prime}}\end{array}\right]$$ with a mean zero and a covariance structure matrix $\mathbf{H}$, $$\left[\begin{array}{c}{\mathbf{u}}_{n}\\ {\mathbf{u}}_{g}\end{array}\right]\sim MVN\left({\mathbf{0}},\mathbf{H}{\sigma }_{u}^{2}\right),$$ where ${\sigma }_{u}^{2}$ is the additive genetic variance and $\mathbf{H}$ is the additive genetic covariance matrix defined below. Without loss of generality, we assume an independent and identical normal distribution for the residual effects $\mathbf{e}\sim MVN\left({\mathbf{0}},\mathbf{I}{\sigma }_{e}^{2}\right),$ where $\mathbf{I}$ is the identity matrix and ${\sigma }_{e}^{2}$ is the residual variance.

In ssGTBLUP [8], the genomic relationship matrix is assumed to have the form ${\mathbf{G}}_{\mathrm{C}}={\mathbf{G}}_{\mathrm{m}}+\mathbf{C},$ where ${\mathbf{G}}_{\mathrm{m}}$ is a function of genomic data and $\mathbf{C}$ is an invertible regularization matrix. We define the genomic part as having the form ${\mathbf{G}}_{\mathrm{m}}=\mathbf{Z}\mathbf{B}\mathbf{Z}^\mathrm{\prime},$ where $\mathbf{Z}=(\mathbf{M}-\mathbf{P})$ is an $n$ by $m$ matrix of centered marker genotypes with $n$ being the number of genotyped animals and $m$ being the number of SNPs, $\mathbf{M}$ is an $n$ by $m$ matrix of SNP genotypes, $\mathbf{P}$ is an $n$ by $m$ centering matrix and $\mathbf{B}$ is an $m$ by $m$ diagonal scaling matrix. The centering matrix has often the form $\mathbf{P}=2{\mathbf{1}}_{n}\mathbf{p}^{\mathbf{\prime}}$ where the vector $\mathbf{p}$ has $m$ allele frequencies. The marker genotype matrix $\mathbf{M}$ has counts of the first allele, such that the homozygous genotype for the second allele has a value of 0, the heterozygous genotype has 1, and the homozygous genotype for the second allele has 2. It is recommended to use base population allele frequencies in the vector $\mathbf{p}$ [14]. In VanRaden’s [14] Method 1, the scaling matrix $\mathbf{B}$ is equal to ${\mathbf{B}}_{VR}=\mathbf{I}\frac{1}{k}$ with the scaling constant $k=2{\sum }_{i=1}^{m}{p}_{i}{(1-p}_{i})$. The linear system of MME for ssGTBLUP is [8]:

2 $$\left[\begin{array}{ccc}{\mathbf{X}}^{\mathbf{\prime}}\mathbf{X}& {\mathbf{X}}_{n}^{\mathbf{\prime}}{\mathbf{W}}_{n}& {\mathbf{X}}_{g}^{\mathbf{\prime}}{\mathbf{W}}_{g}\\ {\mathbf{W}}_{n}^{\mathbf{\prime}}{\mathbf{X}}_{n}& {\mathbf{W}}_{n}^{\mathbf{\prime}}{\mathbf{W}}_{n}+{\mathbf{H}}^{nn}\lambda & {\mathbf{H}}^{ng}\lambda \\ {\mathbf{W}}_{g}^{\mathbf{\prime}}{\mathbf{X}}_{g}& {\mathbf{H}}^{gn}\lambda & {\mathbf{W}}_{g}^{\mathbf{\prime}}{\mathbf{W}}_{g}+{\mathbf{H}}^{gg}\lambda \end{array}\right]\left[\begin{array}{c}\widehat{\mathbf{b}}\\ {\widehat{\mathbf{u}}}_{n}\\ {\widehat{\mathbf{u}}}_{g}\end{array}\right]=\left[\begin{array}{c}{\mathbf{X}}^{\mathbf{\prime}}\mathbf{y}\\ {\mathbf{W}}_{n}^{\mathbf{\prime}}\mathbf{y}\\ {\mathbf{W}}_{g}^{\mathbf{\prime}}\mathbf{y}\end{array}\right],$$

where $\lambda =\frac{{\sigma }_{e}^{2}}{{\sigma }_{u}^{2}}$, $$\mathbf{X}^{\mathbf{\prime}}=\left[\begin{array}{cc}{\mathbf{X}}_{n}^{\mathbf{\prime}}& {\mathbf{X}}_{g}^{\mathbf{\prime}}\end{array}\right]$$, $${\mathbf{H}}^{-1}=\left[\begin{array}{cc}{\mathbf{H}}^{nn}& {\mathbf{H}}^{ng}\\ {\mathbf{H}}^{gn}& {\mathbf{H}}^{gg}\end{array}\right]={\mathbf{A}}^{-1}+\left[\begin{array}{cc}{\mathbf{0}}& {\mathbf{0}}\\ {\mathbf{0}}& {\mathbf{G}}_{C}^{-1}-{\mathbf{A}}_{gg}^{-1}\end{array}\right]$$, **A**^–1^ is the inverse of the pedigree-based relationship matrix, and ${\mathbf{A}}_{gg}$ is the pedigree-based relationship matrix among the genotyped animals. Matrix $${\mathbf{A}}^{-1}=\left[\begin{array}{cc}{\mathbf{A}}^{nn}& {\mathbf{A}}^{ng}\\ {\mathbf{A}}^{gn}& {\mathbf{A}}^{gg}\end{array}\right]$$ is denoted similarly as for ${\mathbf{H}}^{-1}$. The inverse genomic relationship matrix can be expressed using the Woodbury matrix identity [8] as:

$${\mathbf{G}}_{C}^{-1}={\left(\mathbf{Z}\mathbf{B}\mathbf{Z}^\mathrm{\prime}+\mathbf{C}\right)}^{-1}={\mathbf{C}}^{-1}-{\mathbf{C}}^{-1}\mathbf{Z}{\mathbf{K}}^{-1}{\mathbf{Z}}^{\mathbf{\prime}}{\mathbf{C}}^{-1},$$

where $\mathbf{K}={\mathbf{Z}}^{\mathbf{\prime}}{\mathbf{C}}^{-1}\mathbf{Z}+{\mathbf{B}}^{-1}$ is a symmetric positive definite matrix.

Solving MME (2) iteratively using the preconditioned conjugate gradient (PCG) approach requires computing the product of the MME coefficient matrix times a vector, say $\mathbf{v}$. When the number of genotyped animals is large, most of the computing time for this product is due to ${\mathbf{G}}_{C}^{-1}\mathbf{v}$. Thus, it is important that the product ${\mathbf{C}}^{-1}\mathbf{v}$ is fast, especially for many genotyped animals. Mäntysaari et al. [8] presented two previously proposed forms for $\mathbf{C}$ that allow fast computation of ${\mathbf{C}}^{-1}\mathbf{v}.$ First, $\mathbf{C}=\upvarepsilon \mathbf{I},$ where $\upvarepsilon$ is a small number (e.g., 10^–2^). Second, $\mathbf{C}=w{\mathbf{A}}_{gg}$ where $w$ is the proportion of polygenic variance not accounted for by the markers, i.e., the RPG proportion. When $\mathbf{C}=\upvarepsilon \mathbf{I},$ the $\mathbf{K}$ matrix becomes $\mathbf{K}=\frac{1}{\varepsilon }{\mathbf{Z}}^{\mathbf{\prime}}\mathbf{Z}+{\mathbf{B}}^{-1},$ with $\mathbf{B}={\mathbf{B}}_{VR}=\mathbf{I}\frac{1}{k}.$ When $\mathbf{C}=w{\mathbf{A}}_{gg}$, the $\mathbf{K}$ matrix becomes $\mathbf{K}=\frac{1}{w}{\mathbf{Z}}^{\mathbf{\prime}}{\mathbf{A}}_{gg}^{-1}\mathbf{Z}+{\mathbf{B}}^{-1}.$ Furthermore, the proportion ($1-w$) of additive genetic variance accounted for by the markers must be included in the scaling matrix $\mathbf{B}$, i.e., $\mathbf{B}=\left(1-w\right){\mathbf{B}}_{VR}=\mathbf{I}\frac{1-w}{k}$ when ${\mathbf{G}}_{\mathrm{m}}$ is computed following VanRaden’s [14] Method 1.

The first proposition of the ssGTBLUP approach [8] (hereafter called original ssGTBLUP approach) was derived to give the product ${\mathbf{G}}_{C}^{-1}\mathbf{v}$ of form ${\mathbf{G}}_{C}^{-1}\mathbf{v}=\left({\mathbf{C}}^{-1}-{\mathbf{T}}_{\mathrm{C}}^{\mathbf{\prime}}{\mathbf{T}}_{\mathrm{C}}\right)\mathbf{v},$ where ${\mathbf{T}}_{\mathrm{C}}={\mathbf{L}}_{\mathrm{C}}^{-1}{\mathbf{Z}}^{\mathbf{\prime}}{\mathbf{C}}^{-1},$ with the lower triangular matrix ${\mathbf{L}}_{\mathrm{C}}$ being the Cholesky decomposition of $\mathbf{K}$, i.e., ${\mathbf{L}}_{\mathrm{C}}{\mathbf{L}}_{\mathrm{C}}^{\mathbf{\prime}}=\mathbf{K}.$ Using double-precision arithmetic, software that rely on this original ssGTBLUP approach will use 8* nm* bytes for storing ${\mathbf{T}}_{\mathrm{C}}$ in memory.

However, instead of computing the product ${\mathbf{G}}_{C}^{-1}\mathbf{v}=\left({\mathbf{C}}^{-1}-{\mathbf{T}}_{\mathrm{C}}^{\mathbf{\prime}}{\mathbf{T}}_{\mathrm{C}}\right)\mathbf{v}$, a computationally more efficient approach proposed by Mäntysaari et al. [3] can be to use the original form explicitly, as follows (hereafter called component-wise ssGTBLUP approach):

$${\mathbf{G}}_{C}^{-1}\mathbf{v}={\mathbf{C}}^{-1}\mathbf{v}-{\mathbf{C}}^{-1}\left(\mathbf{Z}\left({\mathbf{L}}_{C}\backslash \left\{{\mathbf{L}}_{C}^{\mathbf{\prime}}\backslash \left[{\mathbf{Z}}^{\mathbf{\prime}}\left({\mathbf{C}}^{-1}\mathbf{v}\right)\right]\right\}\right)\right),$$

where the matrix times vector products are calculated from the innermost brackets to outward, and the backslash ($\backslash$) is an operator indicating that the system of equations is solved by forward or backward substitutions. This approach allows the computations that involve $\mathbf{Z}$ to use $\mathbf{M}$, which can be efficiently stored in a compressed form to take less memory than ${\mathbf{T}}_{\mathrm{C}}$ [3].

#### From ssGTBLUP to ssSNPBLUP

The MME (2) can be reformulated using an equivalent model by appending the vector of estimated SNP marker effect solutions $\widehat{\mathbf{g}}$ to the vector of solutions of MME (2) [7]. An extended MME form can be written as:

3 $$\left[\begin{array}{cccc}\mathbf{X}^{\mathbf{\prime}}\mathbf{X}& {\mathbf{X}}_{n}^{\mathbf{\prime}}{\mathbf{W}}_{n}& {\mathbf{X}}_{g}^{\mathbf{\prime}}{\mathbf{W}}_{g}& {\mathbf{0}}\\ {\mathbf{W}}_{n}^{\mathbf{\prime}}{\mathbf{X}}_{n}& {\mathbf{W}}_{n}^{\mathbf{\prime}}{\mathbf{W}}_{n}+{\mathbf{A}}^{nn}\lambda & {\mathbf{A}}^{ng}\lambda & {\mathbf{0}}\\ {\mathbf{W}}_{g}^{\mathbf{\prime}}{\mathbf{X}}_{g}& {\mathbf{A}}^{gn}\lambda & {\mathbf{W}}_{g}^{\mathbf{\prime}}{\mathbf{W}}_{g}+\left({\mathbf{A}}^{gg}-{\mathbf{A}}_{gg}^{-1}+{\mathbf{C}}^{-1}\right)\lambda & -{\mathbf{C}}^{-1}\mathbf{Z}\lambda \\ {\mathbf{0}}& {\mathbf{0}}& -\mathbf{Z}^{\mathbf{\prime}}{\mathbf{C}}^{-1}\lambda & \mathbf{K}\lambda \end{array}\right]\left[\begin{array}{c}\widehat{\mathbf{b}}\\ {\widehat{\mathbf{u}}}_{n}\\ {\widehat{\mathbf{u}}}_{g}\\ \widehat{\mathbf{g}}\end{array}\right]=\left[\begin{array}{c}{\mathbf{X}}^{\mathbf{\prime}}\mathbf{y}\\ {\mathbf{W}}_{n}^{\mathbf{\prime}}\mathbf{y}\\ {\mathbf{W}}_{g}^{\mathbf{\prime}}\mathbf{y}\\ {\mathbf{0}}\end{array}\right],$$

where the vector $\widehat{\mathbf{g}}$ has the estimated SNP effect solutions $.$ The extended MME (3) is the same as that derived for the ssSNPBLUP when the RPG regularization matrix $\mathbf{C}=w{\mathbf{A}}_{gg}$ is used.

We can denote the inverse of the covariance structure in MME (3) as:

$${\mathbf{H}}_{L}^{-1}=\left[\begin{array}{ccc}{\mathbf{A}}^{nn}& {\mathbf{A}}^{ng}& {\mathbf{0}}\\ {\mathbf{A}}^{gn}& {\mathbf{A}}^{gg}-{\mathbf{A}}_{gg}^{-1}+{\mathbf{C}}^{-1}& -{\mathbf{C}}^{-1}\mathbf{Z}\\ {\mathbf{0}}& -\mathbf{Z}{^{\prime}}{\mathbf{C}}^{-1}& \mathbf{K}\end{array}\right].$$

Following Liu et al. [7], we have $$\mathrm{Var}\left(\begin{array}{c}{\mathbf{u}}_{n}\\ {\mathbf{u}}_{g}\\ \mathbf{g}\end{array}\right)={\mathbf{H}}_{L}{\sigma }_{u}^{2}$$ with ${\mathbf{H}}_{L}$ defined as:

$${\mathbf{H}}_{L}=\left[\begin{array}{ccc}{\mathbf{A}}_{nn}+{\mathbf{A}}_{ng}{\mathbf{A}}_{gg}^{-1}\left({\mathbf{G}}_{C}-{\mathbf{A}}_{gg}\right){\mathbf{A}}_{gg}^{-1}{\mathbf{A}}_{gn}& {\mathbf{A}}_{ng}{\mathbf{A}}_{gg}^{-1}{\mathbf{G}}_{C}& {\mathbf{A}}_{ng}{\mathbf{A}}_{gg}^{-1}\mathbf{Z}\mathbf{B}\\ {\mathbf{G}}_{C}{\mathbf{A}}_{gg}^{-1}{\mathbf{A}}_{gn}& {\mathbf{G}}_{C}& \mathbf{Z}\mathbf{B}\\ \mathbf{B}\mathbf{Z}^{\mathbf{\prime}}{\mathbf{A}}_{gg}^{-1}{\mathbf{A}}_{gn}& \mathbf{B}\mathbf{Z}^{\mathbf{\prime}}& \mathbf{B}\end{array}\right]=\left[\begin{array}{ccc}{\mathbf{A}}_{nn}+{\mathbf{A}}_{ng}{\mathbf{A}}_{gg}^{-1}\left(\left({\mathbf{G}}_{m}+\mathbf{C}\right)-{\mathbf{A}}_{gg}\right){\mathbf{A}}_{gg}^{-1}{\mathbf{A}}_{gn}& {\mathbf{A}}_{ng}{\mathbf{A}}_{gg}^{-1}\left({\mathbf{G}}_{m}+\mathbf{C}\right)& {\mathbf{A}}_{ng}{\mathbf{A}}_{gg}^{-1}\mathbf{Z}\mathbf{B}\\ \left({\mathbf{G}}_{m}+\mathbf{C}\right){\mathbf{A}}_{gg}^{-1}{\mathbf{A}}_{gn}& \left({\mathbf{G}}_{m}+\mathbf{C}\right)& \mathbf{Z}\mathbf{B}\\ \mathbf{B}\mathbf{Z}^{\mathbf{\prime}}{\mathbf{A}}_{gg}^{-1}{\mathbf{A}}_{gn}& \mathbf{B}\mathbf{Z}^{\mathbf{\prime}}& \mathbf{B}\end{array}\right]=\left[\begin{array}{ccc}{\mathbf{A}}_{ng}{\mathbf{A}}_{gg}^{-1}{\mathbf{G}}_{m}{\mathbf{A}}_{gg}^{-1}{\mathbf{A}}_{gn}& {\mathbf{A}}_{ng}{\mathbf{A}}_{gg}^{-1}{\mathbf{G}}_{m}& {\mathbf{A}}_{ng}{\mathbf{A}}_{gg}^{-1}\mathbf{Z}\mathbf{B}\\ {\mathbf{G}}_{m}{\mathbf{A}}_{gg}^{-1}{\mathbf{A}}_{gn}& {\mathbf{G}}_{m}& \mathbf{Z}\mathbf{B}\\ \mathbf{B}\mathbf{Z}^{\mathbf{\prime}}{\mathbf{A}}_{gg}^{-1}{\mathbf{A}}_{gn}& \mathbf{B}\mathbf{Z}^{\mathbf{\prime}}& \mathbf{B}\end{array}\right]+\left[\begin{array}{ccc}{\mathbf{A}}_{nn}-{\mathbf{A}}_{ng}{\mathbf{A}}_{gg}^{-1}{\mathbf{A}}_{gn}& {\mathbf{0}}& {\mathbf{0}}\\ {\mathbf{0}}& {\mathbf{0}}& {\mathbf{0}}\\ {\mathbf{0}}& {\mathbf{0}}& {\mathbf{0}}\end{array}\right]+\left[\begin{array}{ccc}{\mathbf{A}}_{ng}{\mathbf{A}}_{gg}^{-1}\mathbf{C}{\mathbf{A}}_{gg}^{-1}{\mathbf{A}}_{gn}& {\mathbf{A}}_{ng}{\mathbf{A}}_{gg}^{-1}\mathbf{C}& {\mathbf{0}}\\ \mathbf{C}{\mathbf{A}}_{gg}^{-1}{\mathbf{A}}_{gn}& \mathbf{C}& {\mathbf{0}}\\ {\mathbf{0}}& {\mathbf{0}}& {\mathbf{0}}\end{array}\right].$$

This is the same definition of the (co)variance structure matrix used in Christensen and Lund [2] when $\mathbf{C}=w{\mathbf{A}}_{gg}$ in ${\mathbf{H}}_{L}.$ When $\mathbf{C}={\mathbf{0}}$ in ${\mathbf{H}}_{L},$ we have the same definition of the (co)variance structure matrix that is used in Legarra et al. [15] and Fernando et al. [6]. Thus, on the one hand, changes in the regularization matrix $\mathbf{C}$ affect the part of the genetic covariance not including the SNP effects. On the other hand, changes in the scaling matrix $\mathbf{B}$ affect only the marker effect (co)variances. Furthermore, it should be noted that the upper left two by two block of matrices in ${\mathbf{H}}_{L}$ is the same as the $\mathbf{H}$ of ssGBLUP. This further illustrates the auxiliary nature of the vector of SNP effects $\mathbf{g}$ in the described ssSNPBLUP. In other words, the breeding values in ssGBLUP and ssSNPBLUP have the same covariance structure, which have been augmented with the marker covariances in ssSNPBLUP. It is worth noting that MME (3) cannot be derived for $\mathbf{C}={\mathbf{0}}$ because $\mathbf{C}={\mathbf{0}}$ is not invertible.

### Indirect prediction of GEBV for genotyped selection candidates

Computation of GEBV for the genotyped selection candidates, i.e., genotyped animals without own and progeny records, using solutions from the previous ssGBLUP evaluation facilitates earlier obtention of selection candidate predictions than waiting for the next full data genetic evaluation. Furthermore, to reduce the computational costs of the single-step genomic evaluations, it can be of interest [16] to ignore the genotypes of animals without own and progeny records and to predict indirectly their GEBV afterwards by using the solutions of the latest single-step genomic evaluation.

Computation of indirect GEBV predictions can use the decomposition of GEBV $$\mathbf{u}^{\mathbf{\prime}}=\left[\begin{array}{cc}{\mathbf{u}}_{n}^{\mathbf{\prime}}& {\mathbf{u}}_{g}^{\mathbf{\prime}}\end{array}\right]$$ corresponding to the (co)variance structure matrix ${\mathbf{H}}_{L}$, as follows:

4 $$\left[\begin{array}{c}{\mathbf{u}}_{n}\\ {\mathbf{u}}_{g}\end{array}\right]=\left[\begin{array}{c}{\mathbf{A}}_{ng}{\mathbf{A}}_{gg}^{-1}\mathbf{Z}\mathbf{g}\\ \mathbf{Z}\mathbf{g}\end{array}\right]+\left[\begin{array}{c}{\varvec{\upepsilon}}\\ {\mathbf{0}}\end{array}\right]+\left[\begin{array}{c}{\mathbf{d}}_{n}\\ {\mathbf{d}}_{g}\end{array}\right],$$

where $$\left[\begin{array}{c}{\mathbf{A}}_{ng}{\mathbf{A}}_{gg}^{-1}\mathbf{Z}\mathbf{g}\\ \mathbf{Z}\mathbf{g}\end{array}\right]\sim MVN\left({\mathbf{0}},\left[\begin{array}{cc}{\mathbf{A}}_{ng}{\mathbf{A}}_{gg}^{-1}{\mathbf{G}}_{m}{\mathbf{A}}_{gg}^{-1}{\mathbf{A}}_{gn}& {\mathbf{A}}_{ng}{\mathbf{A}}_{gg}^{-1}{\mathbf{G}}_{m}\\ {\mathbf{G}}_{m}{\mathbf{A}}_{gg}^{-1}{\mathbf{A}}_{gn}& {\mathbf{G}}_{m}\end{array}\right]{\sigma }_{u}^{2}\right),$$ ${\varvec{\upepsilon}}\sim MVN\left({\mathbf{0}},\left({\mathbf{A}}_{nn}-{\mathbf{A}}_{ng}{\mathbf{A}}_{gg}^{-1}{\mathbf{A}}_{gn}\right){\sigma }_{u}^{2}\right)$ is the vector of imputation residuals, and $$\mathbf{d}=\left[\begin{array}{c}{\mathbf{d}}_{n}\\ {\mathbf{d}}_{g}\end{array}\right]\sim MVN\left({\mathbf{0}},\left[\begin{array}{cc}{\mathbf{A}}_{ng}{\mathbf{A}}_{gg}^{-1}\mathbf{C}{\mathbf{A}}_{gg}^{-1}{\mathbf{A}}_{gn}& {\mathbf{A}}_{ng}{\mathbf{A}}_{gg}^{-1}\mathbf{C}\\ \mathbf{C}{\mathbf{A}}_{gg}^{-1}{\mathbf{A}}_{gn}& \mathbf{C}\end{array}\right]{\sigma }_{u}^{2}\right)$$ is a vector that corresponds to the part of the genetic effects not explained by the genomic data.

If $\mathbf{C}=\upvarepsilon \mathbf{I},$ the $\mathbf{d}$ vector is usually neglected. However, when $\mathbf{C}=w{\mathbf{A}}_{gg}$, the $\mathbf{d}$ vector corresponds to the RPG effects [2] which account for the genetic variation that is not accounted for by the markers. Thus, for the genotyped animals, the GEBV can be decomposed into two components: the direct genetic value due to the marker effects, $\mathbf{Z}\mathbf{g},$ and the estimated breeding value due to the RPG effects ${\mathbf{d}}_{\mathrm{g}}$ [2, 12, 17].

Based on Eq. (4), the GEBV of genotyped selection candidates can be predicted using already computed GEBV ($\widehat{\mathbf{u}}$) and SNP solutions ($\widehat{\mathbf{g}}$) from ssSNPBLUP. GEBV of the genotyped selection candidates consist of two components: the direct genetic value due to the marker effects (i.e., ${\mathbf{Z}}_{c}\widehat{\mathbf{g}}$) and the estimated breeding value due to the RPG effects (i.e., ${\widehat{\mathbf{d}}}_{c}$). Thus, the GEBV of the genotyped selection candidates can be computed as ${\widehat{\mathbf{u}}}_{c}={\mathbf{Z}}_{c}\widehat{\mathbf{g}}+{\widehat{\mathbf{d}}}_{c}$. When ssGTBLUP has been used, the marker solutions $\widehat{\mathbf{g}}$ can be easily calculated in a post-processing step after solving the MME (2) by using the Eq. (17) in Liu et al. [7]:

5 $$\widehat{\mathbf{g}}={\mathbf{K}}^{-1}\mathbf{Z}^{\mathbf{\prime}}{\mathbf{C}}^{-1}{\widehat{\mathbf{u}}}_{g}.$$

The computation of GEBV for the selection candidates (${\widehat{\mathbf{u}}}_{c}$) is straightforward when the model has no RPG effects (i.e., ${\mathbf{d}}_{\mathrm{c}}={\mathbf{0}}$) or if the effect of the regularization matrix $\mathbf{C}$ can be ignored (i.e., ${\mathbf{d}}_{\mathrm{c}}\approx {\mathbf{0}}$). For these cases, the GEBV of the genotyped selection candidates can be directly computed using their centered genotypes and the estimated marker effect solutions, as ${\widehat{\mathbf{u}}}_{c}={\mathbf{Z}}_{c}\widehat{\mathbf{g}}$.

### Efficient computation of the d_c_ effects

While the direct genetic values ${\mathbf{Z}}_{c}\widehat{\mathbf{g}}$ due to the marker effects can be easily computed for the genotyped selection candidates, the estimated breeding values due to the RPG effects ${\widehat{\mathbf{d}}}_{c}$ are not directly available from the solutions of the latest single-step evaluation, and must therefore be computed. When $\mathbf{C}=w{\mathbf{A}}_{gg}$, Liu et al. [13] showed that the RPG effects for the genotyped selection candidates can be computed as:

6 $${\widehat{\mathbf{d}}}_{c}=w{\mathbf{A}}_{cg}{\mathbf{G}}_{C}^{-1}{\widehat{\mathbf{u}}}_{g}={\mathbf{A}}_{cg}{\mathbf{A}}_{gg}^{-1}{\widehat{\mathbf{d}}}_{g},$$

where ${\mathbf{A}}_{cg}$ is the pedigree-based relationship matrix between the genotyped selection candidates and the genotyped animals already included in the latest single-step evaluation, and the RPG effects for the genotyped animals are computed as: ${\widehat{\mathbf{d}}}_{g}={\widehat{\mathbf{u}}}_{g}-\mathbf{Z}\widehat{\mathbf{g}}$.

The computation of ${\widehat{\mathbf{d}}}_{c}$ using Eq. (6) can be done using the Eq. (22) of Fernando et al. [6] as follows:

$$\left[\begin{array}{c}{\mathbf{s}}_{o}\\ {\widehat{\mathbf{d}}}_{c}\end{array}\right]=-{\left[\begin{array}{cc}{\mathbf{A}}^{oo}& {\mathbf{A}}^{oc}\\ {\mathbf{A}}^{co}& {\mathbf{A}}^{cc}\end{array}\right]}^{-1}\left[\begin{array}{c}{\mathbf{A}}^{og}\\ {\mathbf{A}}^{cg}\end{array}\right]{\widehat{\mathbf{d}}}_{g},$$

where $$\left[\begin{array}{ccc}{\mathbf{A}}^{oo}& {\mathbf{A}}^{og}& {\mathbf{A}}^{oc}\\ {\mathbf{A}}^{go}& {\mathbf{A}}^{gg}& {\mathbf{A}}^{gc}\\ {\mathbf{A}}^{co}& {\mathbf{A}}^{cg}& {\mathbf{A}}^{cc}\end{array}\right]={\left[\begin{array}{ccc}{\mathbf{A}}_{oo}& {\mathbf{A}}_{og}& {\mathbf{A}}_{oc}\\ {\mathbf{A}}_{go}& {\mathbf{A}}_{gg}& {\mathbf{A}}_{gc}\\ {\mathbf{A}}_{co}& {\mathbf{A}}_{cg}& {\mathbf{A}}_{cc}\end{array}\right]}^{-1}$$ is the inverse of the pedigree relationship matrix partitioned among the genotyped animals ($g$) included in the latest single-step genomic evaluation, the selection candidates ($c$) and the non-genotyped ancestors ($o$) of the genotyped animals and selection candidates, and ${\mathbf{s}}_{o}$ is the vector of estimated breeding values due to the RPG effects for non-genotyped ancestors of the genotyped animals and selection candidates.

To avoid the solving of a system involving the matrix $$\left[\begin{array}{cc}{\mathbf{A}}^{oo}& {\mathbf{A}}^{oc}\\ {\mathbf{A}}^{co}& {\mathbf{A}}^{cc}\end{array}\right]$$, the computation of ${\widehat{\mathbf{d}}}_{c}$ using Eq. (6) can be done efficiently in two steps. First, calculate $\mathbf{x}={\mathbf{A}}_{gg}^{-1}{\widehat{\mathbf{d}}}_{g}$ for which efficient sparse matrix computations can be used [18]. Second, ${\widehat{\mathbf{d}}}_{c}={\mathbf{A}}_{cg}\mathbf{x}$ can be computed using the algorithm of Colleau [19] that performs the full pedigree-based relationship matrix times vector product, i.e. $$\left[\begin{array}{c}{\mathbf{s}}_{n}\\ {\widehat{\mathbf{d}}}_{g}\\ {\widehat{\mathbf{d}}}_{c}\end{array}\right]=\left[\begin{array}{ccc}{\mathbf{A}}_{nn}& {\mathbf{A}}_{ng}& {\mathbf{A}}_{nc}\\ {\mathbf{A}}_{gn}& {\mathbf{A}}_{gg}& {\mathbf{A}}_{gc}\\ {\mathbf{A}}_{cn}& {\mathbf{A}}_{cg}& {\mathbf{A}}_{cc}\end{array}\right]\left[\begin{array}{c}{\mathbf{0}}\\ \mathbf{x}\\ {\mathbf{0}}\end{array}\right]$$.

The computation of ${\widehat{\mathbf{d}}}_{c}={\mathbf{A}}_{cg}{\mathbf{A}}_{gg}^{-1}{\widehat{\mathbf{d}}}_{g}$ can be further simplified by splitting it into separate computations for two sets of animals. In the computation of ${\widehat{\mathbf{d}}}_{c}={\mathbf{A}}_{cg}\mathbf{x}$ using the algorithm of Colleau [19], it can be noted that ${\widehat{\mathbf{d}}}_{c}$ depends only on the estimated breeding values due to the RPG effects of the genotyped animals and all their ancestors. Thus, first, the vector of estimated breeding values due to RPG for the non-genotyped ancestors of the genotyped animals, ${\widehat{\mathbf{d}}}_{an{c}_{g}}$, can be computed as:

$${\widehat{\mathbf{d}}}_{an{c}_{g}}=-{\left({\mathbf{A}}^{an{c}_{g}, an{c}_{g}}\right)}^{-1}{\mathbf{A}}^{an{c}_{g}, g}{\widehat{\mathbf{d}}}_{g},$$

where the matrix $$\left[\begin{array}{cc}{\mathbf{A}}^{an{c}_{g}, an{c}_{g}}& {\mathbf{A}}^{an{c}_{g}, g}\\ {\mathbf{A}}^{ g,an{c}_{g}}& {\mathbf{A}}^{gg}\end{array}\right]$$ is the inverse of the pedigree relationship matrix among the genotyped animals included in the latest single-step genomic evaluation and all their ancestors ($an{c}_{g}$). Second, the vector of estimated breeding values due to RPG for the genotyped selection candidates, ${\widehat{\mathbf{d}}}_{c}$, can be computed by calculating the parent average of the estimated breeding values due to RPG from the oldest to the youngest animal included in the pedigree of the genotyped selection candidates.

The presented formulas are based on derivations from the MME of the ssSNPBLUP model and therefore yield exact solutions. We introduce a regression-based approach for the RPG part of GEBV ${\widehat{\mathbf{d}}}_{c}$ that allows an even simpler computational approach than the one presented. The RPG term ${\widehat{\mathbf{d}}}_{c}$ can be estimated by the mean of parent RPG effect values. The parent average uses ${\widehat{\mathbf{d}}}_{g}$ for the genotyped animals but values from a regression equation are used for the non-genotyped parents. Thus, for the genotyped parents, the RPG part for the genotyped reference animals is computed as ${\widehat{\mathbf{d}}}_{g}={\widehat{\mathbf{u}}}_{g}-\mathbf{Z}\widehat{\mathbf{g}}$. For the non-genotyped parents, ${\widehat{\mathbf{d}}}_{n}$ is approximated by $\widetilde{{\mathbf{d}}_{n}}=\widehat{a}+\widehat{b}{\widehat{\mathbf{u}}}_{n}$ where the coefficients $\widehat{a}$ and $\widehat{b}$ are estimated by linear regression of ${\widehat{\mathbf{d}}}_{g}$ on ${\widehat{\mathbf{u}}}_{g}$, that is ${\widehat{\mathbf{d}}}_{g}=\widehat{a}+\widehat{b}{\widehat{\mathbf{u}}}_{g}$. Thus, ${\widehat{\mathbf{d}}}_{c}$ is approximated by a parent average using values in the vector $$\widetilde{\mathbf{d}}=\left[\begin{array}{cc}\widetilde{{\mathbf{d}}_{n}}^{\mathbf{\prime}}& {\widehat{\mathbf{d}}}_{g}^{\mathbf{\prime}}\end{array}\right]^{\mathbf{\prime}}$$. Then, GEBV of selection candidates can be calculated as ${\widehat{\mathbf{u}}}_{c}={\mathbf{Z}}_{c}\widehat{\mathbf{g}}+\widetilde{{\mathbf{d}}_{{\varvec{c}}}}$.

### Consideration of other effects in the indirect prediction of GEBV

In addition to the direct genetic values due to the marker effects (${\mathbf{Z}}_{c}\widehat{\mathbf{g}}$) and the estimated breeding values due to the RPG effects (${\widehat{\mathbf{d}}}_{c}$) when $\mathbf{C}=w{\mathbf{A}}_{gg}$, the GEBV may also include other effects, such as the contributions of a covariate that models the difference from the pedigree base to the genomic base (hereinafter called J-factor; [20–22]), or the contributions of genetic groups in the model. For example, if a J-factor is fitted in the model, Eq. (4) for GEBV becomes:

$$\left[\begin{array}{c}{\mathbf{u}}_{j,n}\\ {\mathbf{u}}_{j,g}\end{array}\right]=\left[\begin{array}{c}-{\mathbf{A}}_{ng}{\mathbf{A}}_{gg}^{-1}\\ -\mathbf{I}\end{array}\right]{\mathbf{1}}\mu +\left[\begin{array}{c}{\mathbf{A}}_{ng}{\mathbf{A}}_{gg}^{-1}\\ \mathbf{I}\end{array}\right]\mathbf{Z}\mathbf{g}+\left[\begin{array}{c}{\varvec{\upepsilon}}\\ {\mathbf{0}}\end{array}\right]+\left[\begin{array}{c}{\mathbf{d}}_{n}\\ {\mathbf{d}}_{g}\end{array}\right],$$

where ${\mathbf{1}}$ is a vector of 1s, and $\mu$ is the covariate that models the difference from the pedigree base to the genomic base. The GEBV for the genotyped selection candidates can therefore be computed as:

$${\widehat{\mathbf{u}}}_{j,c}=-{1}_{c}\widehat{\mu }+{\mathbf{Z}}_{c}\widehat{\mathbf{g}}+{\widehat{\mathbf{d}}}_{g},$$

with $\widehat{\mu }$ being the solution estimated in the latest single-step genomic evaluation. This approach can also be used for genetic groups.

It is worth noting that our proposed indirect approach is similar to previously proposed indirect approaches (e.g., [11, 12, 23]**)**. However, our proposed approach is different in the sense that all GEBV components are computed without approximation for the indirect computation of GEBV for selection candidates. Further details on the similarities and differences between our approach and previously proposed approaches can be found in the "Discussion" section.

### Data and models

The single-step genomic evaluations and indirect prediction approaches were tested using data from the routine six-trait calving-difficulty evaluation for Irish dairy and beef cattle performed by Irish Cattle Breeding Federation (ICBF; Ireland) in March 2022. The single-step genomic evaluations were based on the same multi-trait animal model and variance components as the current official routine breeding value evaluation described in more detail in Evans et al. [24].

After extraction and editing, the data file included 16.59 million data records (across the 6 traits), and the pedigree included 26.46 million animals. The number of records per trait is in Table 1. The genotypes of 2.61 million genotyped animals included 47,006 SNPs on 29 bovine autosomes, with a minor allele frequency higher or equal to 0.01. The genotype data was from 30 different arrays ranging in size from 3 to 850K SNPs. However, 91% were from the International Beef and Dairy (IDB) customised chip with an array density between 50 and 54K and within those IDB chips 55% were Illumina Bead Chip technology (Illumina, San Diego, USA) and the remaining 45% were Thermofisher Scientific Microarray technology (Thermofisher Scientific Waltham, MA, USA). Missing SNP genotypes were imputed using FImpute [25] to a 50K SNP set based on version 3 of the IDB chip. Among the 2.61 million genotyped animals, 457,171 genotyped animals were without their own and progeny records. The remaining 2.16 million genotyped animals had either their own records or descendants with records.

**Table 1:** Number, means and standard deviations (SD) of records, and heritabilities for the six traits

Trait	Number of records	Mean	SD	${h}_{d}^{2}$	${h}_{m}^{2}$
1	2,099,743	1.30	0.60	0.13	0.04
2	7,085,063	1.20	0.50	0.07	0.02
3	984,905	1.39	0.71	0.16	0.08
4	6,255,183	1.26	0.57	0.14	0.08
5	2,280,213	3.16	0.68	0.19	0.05
6	232,582	41.51	7.94	0.14	0.03

^1^${h}_{d}^{2}$ = heritability of the direct additive genetic effect

^2^${h}_{m}^{2}$ = heritability of the maternal additive genetic effect

The six-trait linear mixed effects model included random effects (additive direct and maternal genetic, contemporary group, and residual effects), fixed co-variables for direct breed proportion (n = 22), dam breed proportion (n = 22), specific heterosis coefficients (n = 13), age of dam (primiparous), age nested with parity (multiparous), and fixed cross-classified effects for birth year and sex of calf. For all single-step genomic evaluations, an additional J-factor, which is a fixed covariable that models the difference from the pedigree base to the genomic base, was fitted separately for the direct and maternal genetic effects of each trait [26]. A single J-factor was fitted for all breeds following Aldridge et al. [27]. The genotype matrix was centred using observed allele frequencies computed over all breeds.

### Study design

#### Single-step genomic evaluations

The ssGTBLUP (hereinafter called ssGTABLUP when an RPG effect is fitted) and ssSNPBLUP models used an RPG effect. The RPG proportion was equal to $w=0.20$. The two models were used to compute GEBV from a full and a reduced dataset. First, the models were solved with the full dataset that included all phenotypic and genomic information, i.e., 2.61 million genotypes (i.e., including genotypes of the selection candidates). Second, both models were solved with a reduced dataset that did not include genotypes of the selection candidates, i.e., there were 2.16 million genotypes. Thus, the reduced dataset analysis included all phenotypic information, but only the genotypes of animals with their own records or with descendants (across all generations) with records. For both datasets, the pedigree was extracted for the phenotyped animals and the selected set of genotyped animals.

All models were solved with the software MiXBLUP 3.0 [28] using the solver hpblup, which used the PCG method for solving the MME. The convergence efficiency of the PCG method relies mainly on the so-called preconditioner $\mathbf{P}$. In this study, for both ssGTABLUP (MME (2)) and ssSNPBLUP (MME (3)), the submatrix of $\mathbf{P}$ corresponding to the fixed effects, ${\mathbf{P}}_{ff}$, was equal to ${\mathbf{P}}_{ff}={\mathbf{X}}^{\mathbf{\prime}}\mathbf{X}+diag\left({\mathbf{X}}^{\mathbf{\prime}}\mathbf{X}\right)\mathbf{*}{10}^{-4}$ with $diag\left({\mathbf{X}}^{\mathbf{\prime}}\mathbf{X}\right)$ corresponding to the diagonal elements of ${\mathbf{X}}^{\mathbf{\prime}}\mathbf{X}$. This addition to the diagonal elements ensures that ${\mathbf{P}}_{ff}$ is positive definite, as required for its Cholesky decomposition. The submatrix of $\mathbf{P}$ corresponding to the random effects, ${\mathbf{P}}_{rr}$, included for both ssGTABLUP and ssSNPBLUP a block-diagonal matrix with blocks corresponding to equations for different traits within a level (e.g., an animal). While the original and component-wise ssGTABLUP approaches have the same coefficient matrix (MME (2)), the block-diagonal matrix of ${\mathbf{P}}_{rr}$ corresponding to ${\mathbf{u}}_{g}$ were different for these two approaches because the contributions of the diagonal elements of ${\frac{1}{{w}^{2}}\mathbf{A}}_{gg}^{-1}\mathbf{Z}{\mathbf{K}}^{-1}\mathbf{Z}^{\mathbf{\prime}}{\mathbf{A}}_{gg}^{-1}$ (being a term of ${\mathbf{G}}_{a}^{-1}-{\mathbf{A}}_{gg}^{-1}={\left(\frac{1}{w}-{\mathbf{1}}\right)\mathbf{A}}_{gg}^{-1}-{\frac{1}{{w}^{2}}\mathbf{A}}_{gg}^{-1}\mathbf{Z}{\mathbf{K}}^{-1}\mathbf{Z}^{\mathbf{\prime}}{\mathrm{A}}_{gg}^{-1}$) were not computed and, thus, were not available for inclusion in for the component-wise ssGTABLUP approach. For ssSNPBLUP, it is worth noting that, for the SNP effects, the *i*-th diagonal element of $\mathbf{Z}^{\mathbf{\prime}}{\mathbf{A}}_{gg}^{-1}\mathbf{Z}$ of $\mathbf{K}=\frac{1}{w}\mathbf{Z}^{\mathbf{\prime}}{\mathbf{A}}_{gg}^{-1}\mathbf{Z}+{\mathbf{B}}^{-1}$ was approximated by $2{p}_{i}{(1-p}_{i})n$ [29], and that a second-level diagonal preconditioner was also included, as in Vandenplas et al. [29].

The software MiXBLUP supports reading genomic information in the Plink 1 binary form [30], and for both the full and reduced datasets the genotypes were provided in this form. Both the original and component-wise approaches for solving ssGTABLUP with an RPG effect are implemented in MiXBLUP. Briefly, for the original ssGTABLUP approach, the solver hpblup requires a matrix equal to ${\mathbf{T}}_{a}=\frac{1}{w}{\mathbf{L}}^{-1}\mathbf{Z}^{\mathbf{\prime}}{\mathbf{A}}_{gg}^{-1}$ with $\mathbf{L}$ being the Cholesky decomposition of $\mathbf{K}=\frac{1}{w}\mathbf{Z}^{\mathbf{\prime}}{\mathbf{A}}_{gg}^{-1}\mathbf{Z}+{\mathbf{B}}^{-1}$. The ${\mathbf{T}}_{a}$ matrix was computed using double-precision arithmetic with the program calc_grm [31], i.e., ${\mathbf{T}}_{a}$ used 8*nm* bytes. In the solving phase, ${\mathbf{T}}_{a}$ was stored in the random access memory (RAM) to allow efficient parallel computations using multi-threading. For the component-wise ssGTABLUP approach, the solver hpblup only requires $\mathbf{L}$ and $\mathbf{M}$, both stored in memory. The marker matrix $\mathbf{M}$ is stored in RAM using the Plink 1 binary form that requires *nm*/4 bytes [26].

For both ssGTABLUP approaches, the SNP effects $\widehat{\mathbf{g}}$ were computed by the solver after the end of the PCG iterative process using ${\mathbf{T}}_{a}$ as $\widehat{\mathbf{g}}={\mathbf{L}}^{-1\mathrm{^{\prime}}}{\mathbf{T}}_{a}{\widehat{\mathbf{u}}}_{g}$ (derived from Eq. (5)) for the original approach, and as $\widehat{\mathbf{g}}=\frac{1}{w}{\mathbf{L}}^{-1\mathrm{^{\prime}}}{\mathbf{L}}^{-1}\mathbf{Z}^{\mathbf{\prime}}{\mathbf{A}}_{gg}^{-1}{\widehat{\mathbf{u}}}_{g}$ for the component-wise approach. This strategy allows the computation of SNP effects in less time than needed for one PCG iteration.

Similarly to the component-wise ssGTABLUP approach, the ssSNPBLUP approach allows direct use of the marker matrix $\mathbf{M}$ for the multiplication of the coefficient matrix by a vector in MME (3). Consequently, for solving the ssSNPBLUP model, the marker matrix $\mathbf{M}$ was also stored in the RAM using the Plink 1 binary form [26].

The convergence criterion for the PCG iteration was the relative difference between the left- and right-hand sides of the MME:

$${\mathrm{C}}_{r}=\sqrt{\frac{\left({\mathbf{C}}_{\mathrm{MME}}{\mathbf{s}}^{[k]}-{\mathbf{r}}_{\mathrm{MME}}\right)^{\mathbf{\prime}}\left({\mathbf{C}}_{\mathrm{MME}}{\mathbf{s}}^{[k]}-{\mathbf{r}}_{\mathrm{MME}}\right)}{{\mathbf{r}}_{\mathrm{MME}}{\mathbf{^\prime}}{\mathbf{r}}_{\mathrm{MME}}},}$$

where ${\mathbf{C}}_{\mathrm{MME}}$ is the coefficient matrix of the MME, ${\mathbf{s}}^{[k]}$ is the vector of solutions at round $k$, and ${\mathbf{r}}_{\mathrm{MME}}$ is the right-hand side vector. For all evaluations, convergence was assumed to be reached when C_r_ < 10^–7^.

#### Indirect approaches

Four approaches for computing indirectly GEBV for the genotyped selection candidates using solutions from the reduced data single-step evaluations were compared with those calculated in the full data single-step evaluations. The indirect prediction approaches were: (1) the parent average (PA): mean of parent GEBV computed in a previous single-step approach; (2) the direct genomic values (DGV) computed as ${\mathbf{Z}}_{c}\widehat{\mathbf{g}}$, using estimated SNP effects $\widehat{\mathbf{g}}$ from the reduced data single-step evaluation; (3) the regression approach (REG): GEBV computed as ${\widetilde{{\varvec{u}}}}_{j,c}=-{\mathbf{1}}\widehat{\mu }+{\mathbf{Z}}_{c}\widehat{\mathbf{g}}+{\widetilde{{\varvec{d}}}}_{{\varvec{c}}}$ with ${\widetilde{{\varvec{d}}}}_{{\varvec{c}}}$ being approximated using the regression approach; and (4) exact computation of GEBV (that is, of genomic and residual polygenic values; GRV): the GEBV computed as ${\widehat{\mathbf{u}}}_{j,c}=-{\mathbf{1}}\widehat{\mu }+{\mathbf{Z}}_{c}\widehat{\mathbf{g}}+{\mathbf{A}}_{cg}{\mathbf{A}}_{gg}^{-1}{\widehat{\mathbf{d}}}_{g} \mathrm{with }{\widehat{\mathbf{d}}}_{g}={\widehat{\mathbf{u}}}_{g}-\mathbf{Z}\widehat{\mathbf{g}}.$

The PA approach is the simplest approach and can be considered as a reference. The DGV, REG, and GRV approaches were implemented in a Fortran 2018 program called indirectpred. This program requires the genotypes, the inbreeding coefficients, the SNP effect solutions, and the GEBV solutions for all animals included in the reduced data single-step evaluation, as well as the genotypes and the pedigree of the genotyped selection candidates.

GEBV computed indirectly for the genotyped selection candidates were compared with the GEBV computed from the full data single-step evaluations for both ssSNPBLUP and ssGTABLUP. For each trait and for both direct and maternal genetic effects, we calculated (1) the Pearson correlations between GEBV from the full data evaluations and the indirect GEBV hereinafter also called accuracy, (2) dispersion biases as the regression coefficients of the regression of GEBV from the full data evaluations on the indirect GEBV, and (3) level biases for each trait $j$, defined as the average of $\left({\widehat{\mathbf{u}}}_{j,c}-{\widehat{\mathbf{u}}}_{j,c,full}\right)/{\sigma }_{j}$ where ${\widehat{\mathbf{u}}}_{j,c}$ and ${\widehat{\mathbf{u}}}_{j,c,full}$ are the indirect GEBV and the full data GEBV for the genotyped selection candidates, respectively, and ${\sigma }_{j}$ is the genetic standard deviation of trait $j$.

All computations for ssSNPBLUP, ssGTABLUP and indirect approaches, were performed on a computer with 2.9 TB RAM and running RedHat 7.7 (x86_64) with four Intel Xeon Gold 6242 (2.80 GHz) processors, each having 16 cores. The number of OpenMP threads used for all computations was equal to 10. All reported times are indicative, because they may have been influenced by other jobs running simultaneously on the computer.

## Results

### Performances of ssSNPBLUP and ssGTABLUP

The Pearson correlations for all traits between GEBV for ssSNPBLUP and ssGTABLUP were higher than 0.997, and all regression coefficients of the regression of GEBV for ssSNPBLUP on GEBV for ssGTABLUP were between 0.991 and 1.009, for both the reduced and full datasets. Thus, the GEBV for all direct and maternal traits of the ssSNPBLUP and ssGTABLUP evaluations were (almost) the same after convergence was reached for both the reduced and full datasets. Comparing GEBV obtained with the full dataset and the reduced dataset for animals present in both datasets resulted in Pearson correlations higher than 0.993 and regression coefficients of the regression of GEBV for the full dataset on GEBV of the reduced dataset between 0.991 and 1.014, for both ssGTABLUP and ssSNPBLUP (results not shown).

**Fig. 1:**
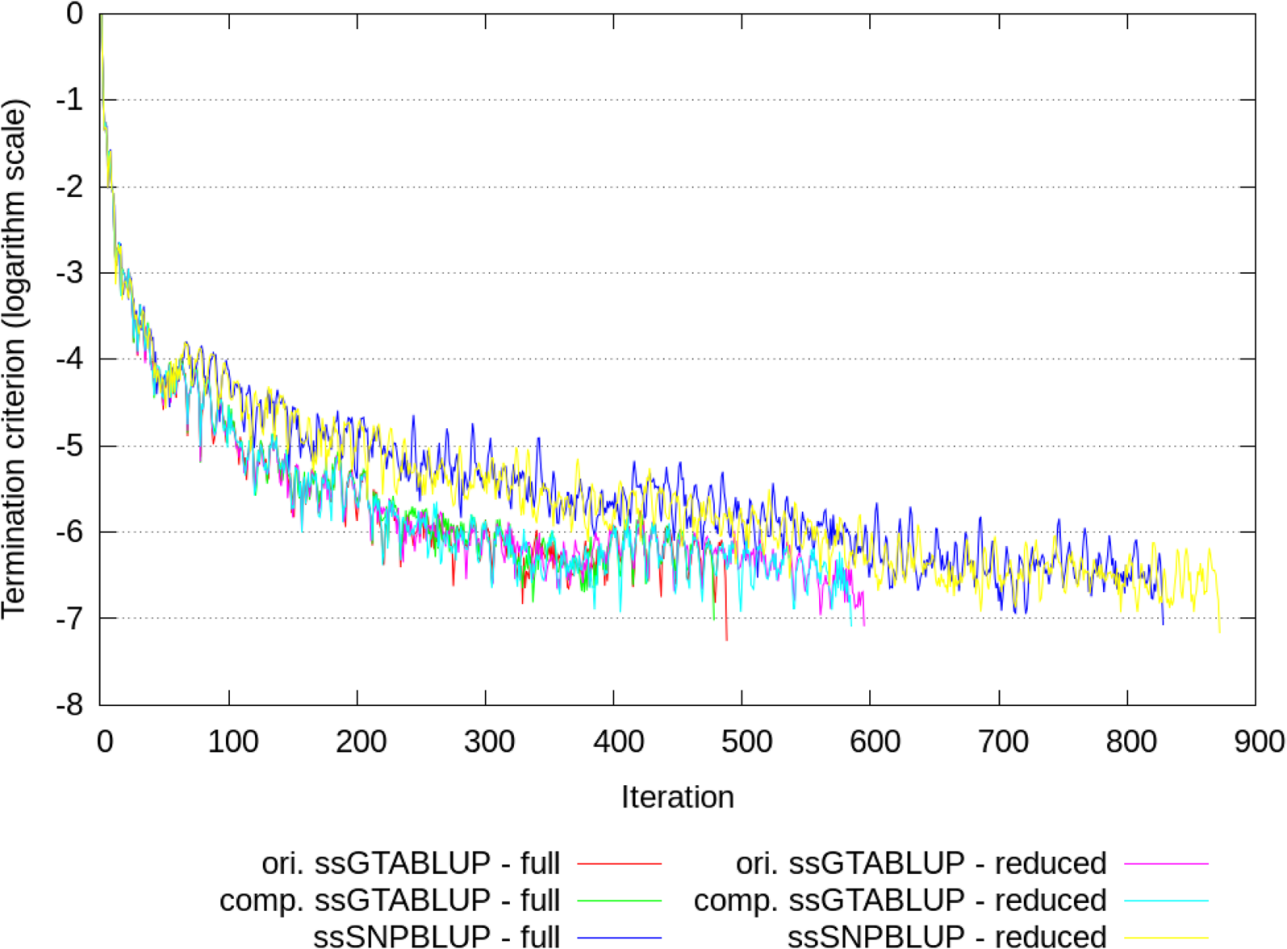
Convergence according to the termination criteria used (y axis in in log_10_ units) during PCG iteration for ssSNBPLUP and the original (ori.) and component-wise (comp.) ssGTABLUP approaches with the reduced and full datasets

Computational statistics of ssGTABLUP and ssSNPBLUP using the full and reduced datasets are in Table 2. The MME of ssGTABLUP and ssSNPBLUP evaluations included about 372 million and 366 million equations with the full and reduced datasets, respectively. When analysing the full dataset, the solver required 1015 GB of RAM and 26 h with 488 iterations for the original ssGTABLUP, 102 GB of RAM and 10 h with 478 iterations for the component-wise ssGTABLUP, and 86 GB RAM and 16 h with 828 iterations for ssSNBPLUP (Fig. 1 and Table 2). Each iteration required on average 172 s for the original ssGTABLUP, and 65 s for both the component-wise ssGTABLUP and ssSNPBLUP. Analysing the reduced dataset resulted in a reduction of the computing time per iteration of about 17% for the original ssGTABLUP, and between 20 and 21% for the component-wise ssGTABLUP and ssSNPBLUP, although the number of iterations to reach convergence increased for both approaches with the reduced dataset. The estimated effective smallest eigenvalues were around 10^–5^ for all systems of equations, while the estimated effective largest eigenvalues were equal to 2.98 for both ssGTABLUP, and a bit larger for ssSNPBLUP (i.e., 3.34 and 3.59 for the reduced and full dataset, respectively; Table 2).

**Table 2:** Computational statistics of ssGTABLUP and ssSNPBLUP using the full and reduced datasets

Computational statistic	Original ssGTABLUP		Component-wise ssGTABLUP		ssSNPBLUP	
	Full	Reduced	Full	Reduced	Full	Reduced
T_a_ matrix (or its components)	1004	832	1000	829	–	–
RAM	31.0	23.6	22.3	18.1	–	–
^a^Time (h)						
Neq	372	366	372	366	372	367
Number of PCG iterations	488	595	478	584	828	872
${\lambda }_{min}$(10^–5^)	4.77	3.13	4.98	3.24	3.42	2.75
${\lambda }_{max}$	2.98	2.98	2.98	2.98	3.59	3.34
RAM^2^ (GB)	1015	845	102	95	86	79
Time/iteration (sec)	172.4	143.4	64.9	51.2	65.4	52.7
^b^Time (h)	26.4	25.9	9.9	9.5	16.4	13.7
Total time (h)	62.2	52.8	37.0	32.5	21.9	18.8

*Neq* = number of equations in millions; *RAM* = software peak random access memory (RAM) defined as the peak resident set size (VmHWM) obtained from the Linux/proc virtual file system; ${\lambda }_{min}$ = smallest effective eigenvalue of the preconditioned coefficient matrix; ${\lambda }_{max}$ = largest effective eigenvalue of the preconditioned coefficient matrix; Time/iteration = Average wall clock time per PCG iteration (expressed in seconds); Total time = wall clock time of the MiXBLUP software expressed in hours

^a^Time = wall clock time of the program calc_grm expressed in hours

^b^Time = wall clock time of the solver expressed in hours

Our implementation of the original ssGTABLUP requires the computation of an additional matrix based on the genomic and pedigree information, that is ${\mathbf{T}}_{a}$. The computation of ${\mathbf{T}}_{a}$ was performed with the program calc_grm and required 1004 GB and 31 h for the full dataset, and 832 GB and 24 h for the reduced dataset (Table 2). Our implementation of the component-wise ssGTABLUP requires the computation of $\mathbf{L}$. Like the computation of ${\mathbf{T}}_{a}$, the computation of $\mathbf{L}$ was performed with the program calc_grm and required 1000 GB and 22 h for the full dataset, and 829 GB and 18 h for the reduced dataset (Table 2). Finally, the complete evaluation that included, among others, the editing and renumbering of all files, the computation of pedigree-based inbreeding coefficients, the computation of the additional matrices for the two ssGTABLUP approaches, and solving of the MME, required 62 and 53 h for the full and reduced original ssGTABLUP, respectively, 37 and 33 h for the full and reduced component-wise ssGTABLUP, respectively, and 22 and 19 h for the full and reduced ssSNPBLUP, respectively. Thus, the reduction in total computing time was around 15% for both ssGTABLUP and ssSNPBLUP when analysing the reduced instead of the full data (Table 2). However, the reduction for the solver step for both ssGTABLUP approaches was almost nil, due to the increase in the number of iterations to reach convergence.

### Accuracies and bias of the different indirect prediction approaches

The indirect predictions of direct and maternal GEBV for all genotyped selection candidates required about 50 GB RAM and 0.5 h for both the original ssGTABLUP and ssSNPBLUP (Table 3). Results for the component-wise ssGTABLUP are equivalent to those obtained with the original ssGTABLUP, because both ssGTABLUP rely on the same MME, and thus are not presented.

**Table 3:** Computational statistics of indirect predictions of direct and maternal GEBV

Model	Approach	RAM (GB)	Total Time (h)
ssGTABLUP	REG	44	0.49
	GRV	50	0.55
ssSNPBLUP	REG	44	0.48
	GRV	50	0.52

*REG.* GEBV with approximated residual polygenic effects, *GRV* exact computation of GEBV. RAM = software peak random access memory (RAM) defined as the peak resident set size (VmHWM) obtained from the Linux/proc virtual file system

**Fig. 2:**
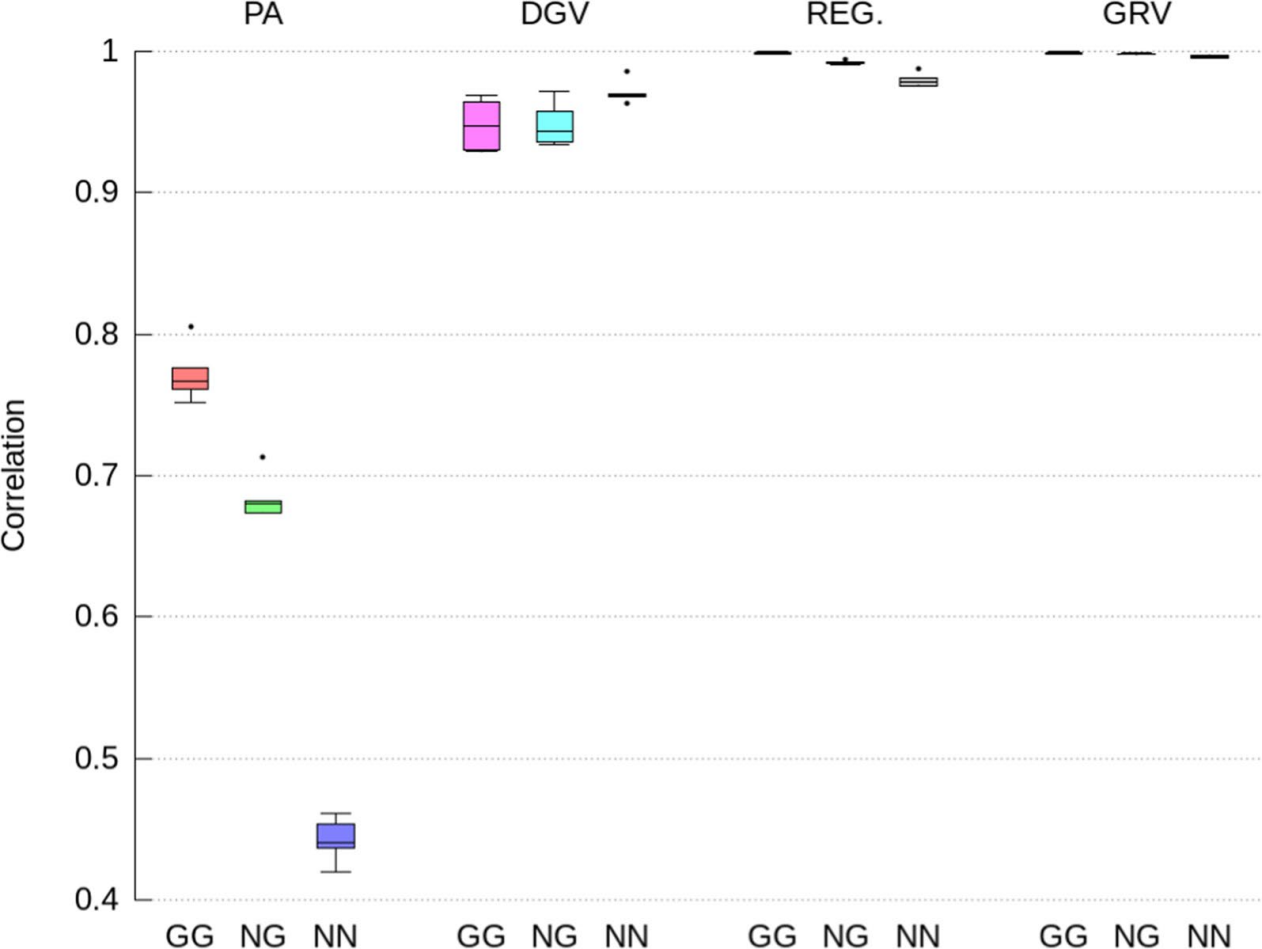
Pearson correlations for direct GEBV computed from the full ssSNPBLUP and from the indirect prediction approaches for genotyped selection candidates with both parents genotyped (GG), with only one parent genotyped (NG), and no parents genotyped (NN). Indirect prediction approaches are: (1) PA: mean of parent GEBV; (2) DGV: direct genomic values; (3) REG: GEBV with approximated residual polygenic effects; and (4) GRV: exact computation of GEBV

**Fig. 3:**
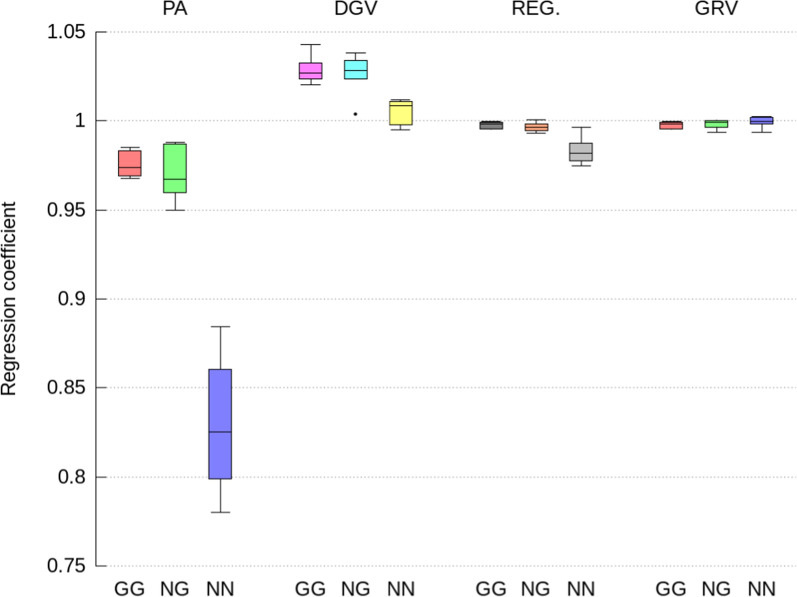
Regression coefficients of direct GEBV computed from the full ssSNPBLUP on GEBV computed from the indirect prediction approaches for genotyped selection candidates with both parents genotyped (GG), with only one parent genotyped (NG), and no parents genotyped (NN). Indirect prediction approaches are: (1) PA: mean of parent GEBV; (2) DGV: direct genomic values; (3) REG: GEBV with approximated residual polygenic effects; and (4) GRV: exact computation of GEBV

**Fig. 4:**
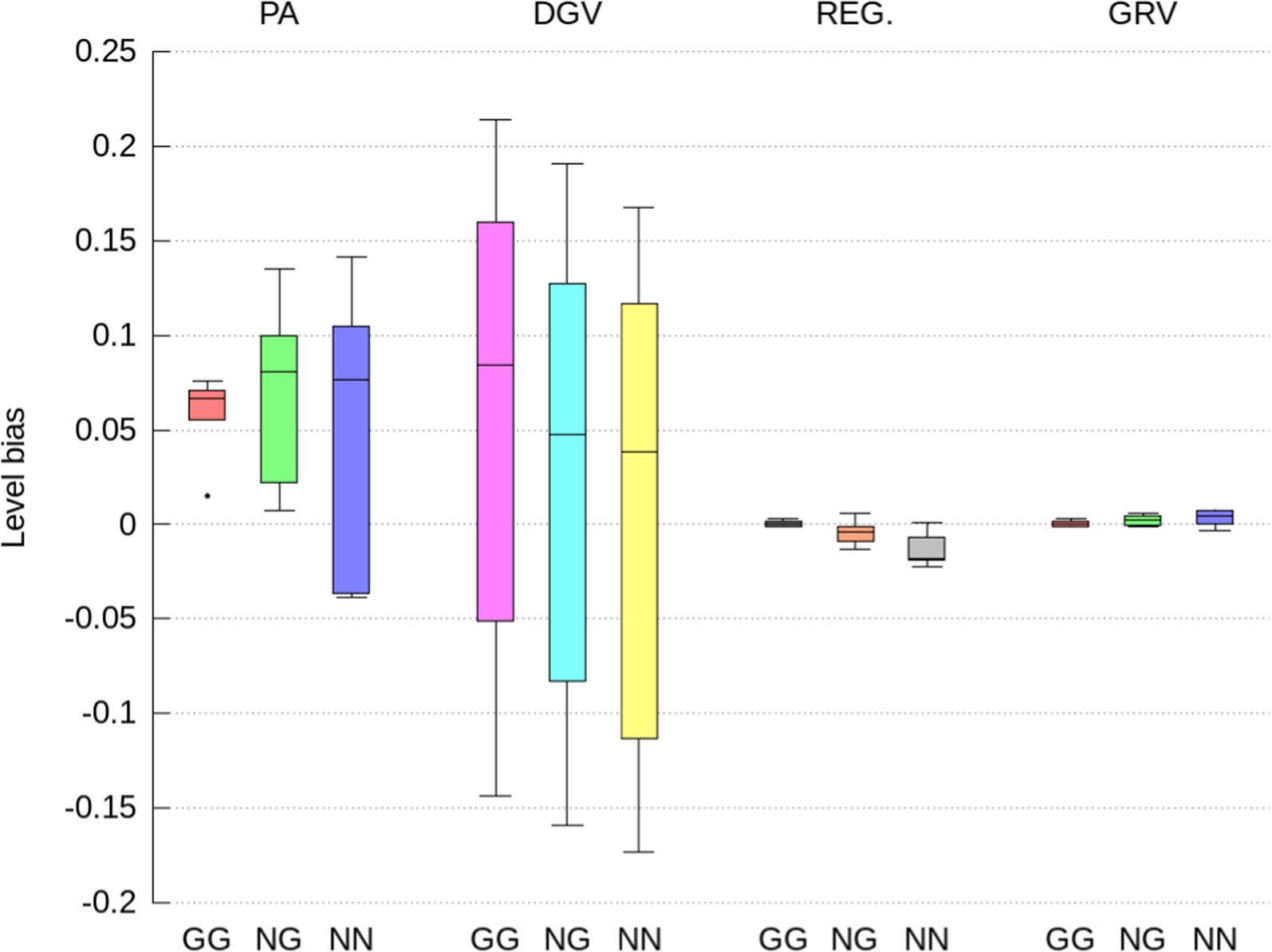
Level bias of direct GEBV computed as the difference between the average of the indirect predictions and ssSNPBLUP solutions expressed in genetic standard deviation units, for genotyped selection candidates with both parents genotyped (GG), with only one parent genotyped (NG), and no parents genotyped (NN). Indirect prediction approaches are: (1) PA: mean of parent GEBV; (2) DGV: direct genomic values; (3) REG: GEBV with approximated residual polygenic effects; and (4) GRV: exact computation of GEBV

Accuracies, dispersion biases, and level biases of the indirect GEBV for the selection candidates with both, only one and no parents genotyped, were computed for both the direct and maternal genetic effects and all traits separately (Figs. 2, 3, 4) and (see Additional file 1: Tables S1 to S6). Because ssGTABLUP and ssSNPBLUP arrived at (almost) the same GEBV, the results for indirect GEBV were also (almost) the same for ssGTABLUP and ssSNPBLUP. Therefore, this section will only present results for the indirect GEBV computed from ssSNPBLUP. All results for both ssGTABLUP and ssSNPBLUP are in Additional file 1: Tables S1–S6.

**Fig. 5:**
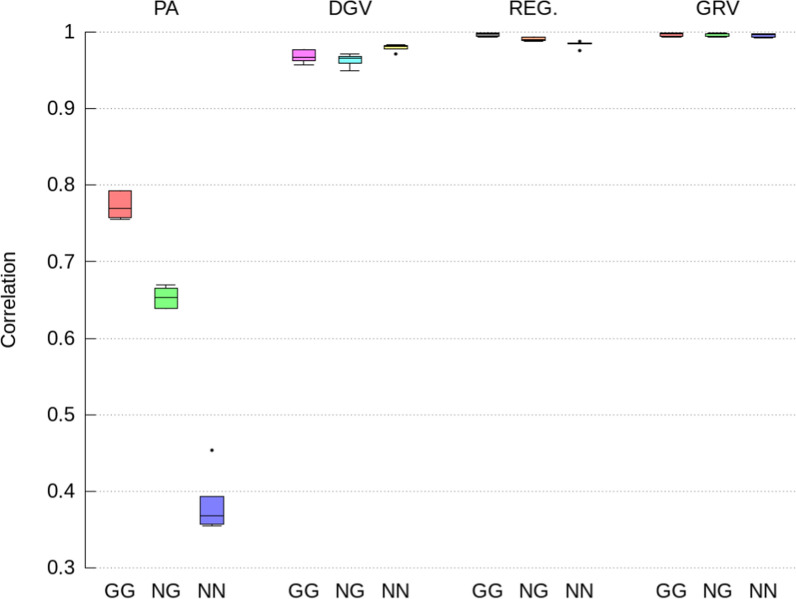
Pearson correlations for maternal GEBV computed from the full ssSNPBLUP and from the indirect prediction approaches for genotyped selection candidates with both parents genotyped (GG), with only one parent genotyped (NG), and no parents genotyped (NN). Indirect prediction approaches are: (1) PA: mean of parent GEBV; (2) DGV: direct genomic values; (3) REG: GEBV with approximated residual polygenic effects; and (4) GRV: exact computation of GEBV

**Fig. 6:**
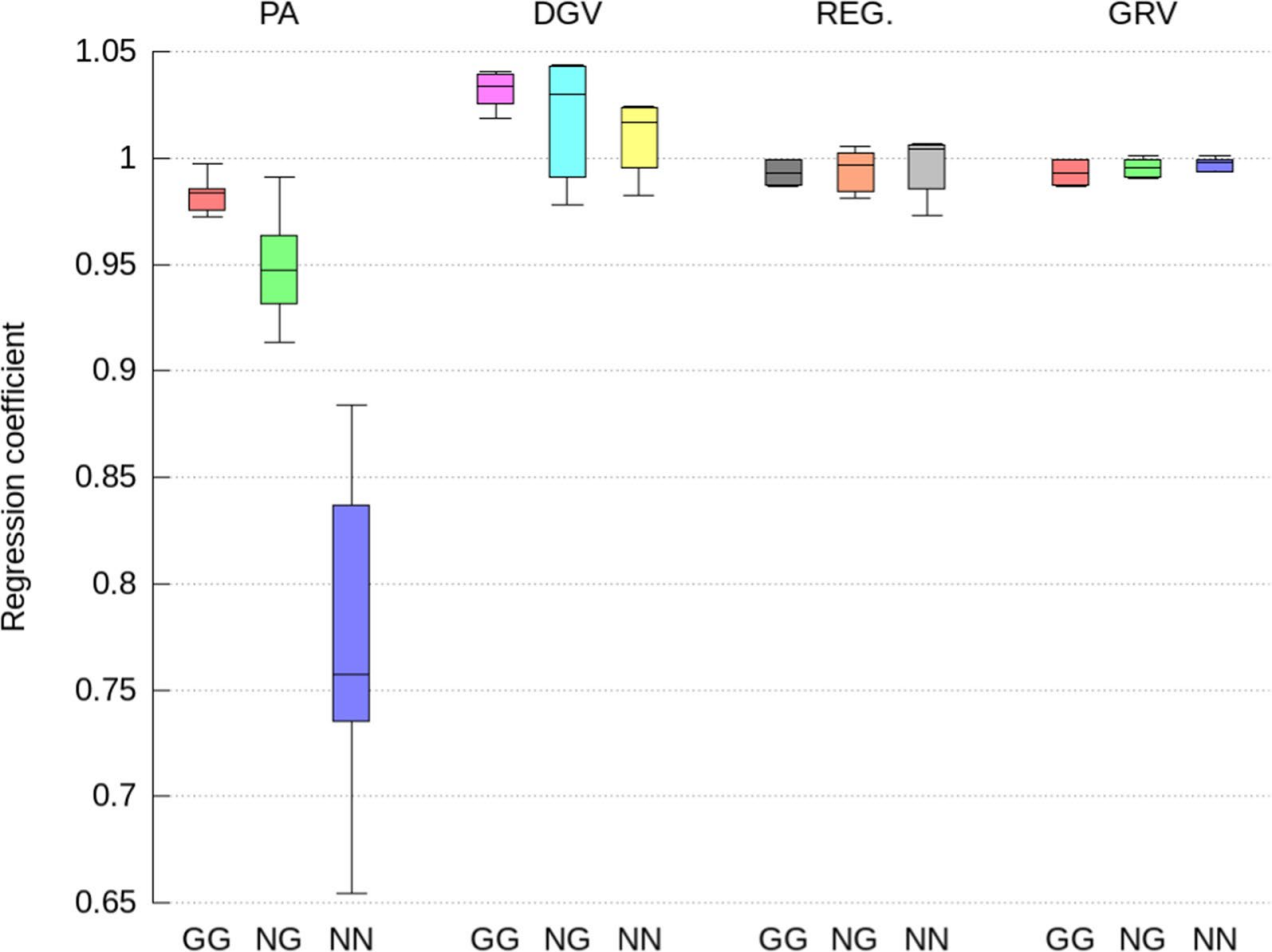
Regression coefficients of maternal GEBV computed from the full ssSNPBLUP on GEBV computed from the indirect prediction approaches for genotyped selection candidates with both parents genotyped (GG), with only one parent genotyped (NG), and no parents genotyped (NN). Indirect prediction approaches are: (1) PA: mean of parent GEBV; (2) DGV: direct genomic values; (3) REG: GEBV with approximated residual polygenic effects; and (4) GRV: exact computation of GEBV

**Fig. 7:**
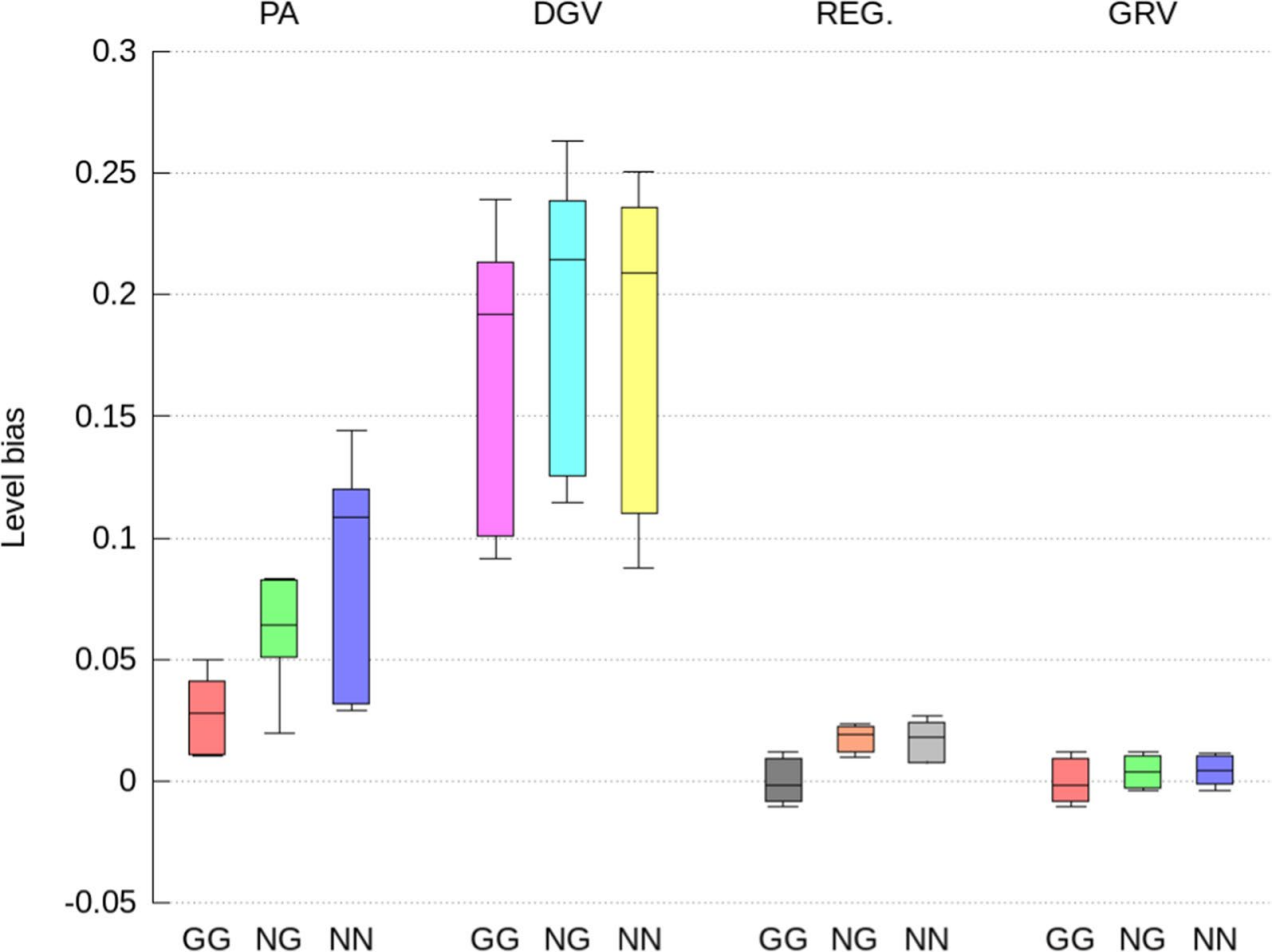
Level bias of maternal GEBV computed as the difference between the average of the indirect predictions and ssSNPBLUP solutions expressed in genetic standard deviation units, for genotyped selection candidates with both parents genotyped (GG), with only one parent genotyped (NG), and no parents genotyped (NN). Indirect prediction approaches are: (1) PA: mean of parent GEBV; (2) DGV: direct genomic values; (3) REG.: GEBV with approximated residual polygenic effects; and (4) GRV: exact computation of GEBV

The indirect GEBV approximated by the GRV approach were associated with the highest accuracies (i.e., correlations higher than 0.996 on average), the lowest level biases, and no over- or under-dispersion (i.e., regression coefficient close to 1.0), across all traits, all genetic effects, and all categories of selection candidates (Figs. 2, 3, 4, 5, 6 and 7). In comparison, the REG approach resulted in the same accuracy and bias as the GRV approach for the selection candidates with both parents genotyped, as expected. However, for the selection candidates with only one or no genotyped parents, indirect GEBV computed with the REG approach for both genetic effects were slightly less accurate (correlations between 0.980 and 0.992 on average), with some dispersion bias (regression coefficients between 0.983 and 0.997), and with some level bias between − 0.014 and 0.018 points of genetic standard deviation (Figs. 2, 3, 4, 5, 6 and 7).

Approximating the GEBV of selection candidates with the DGV approach resulted in indirect GEBV associated with an average accuracy of 0.948 or higher across all groups of selection candidates (Figs. 2 and 5). In addition to being less accurate than the GRV and REG approaches, the indirect GEBV computed with the DGV approach showed also more level bias, which was almost equal to 0.2 points of genetic standard deviation across all traits (Figs. 4 and 7), and more dispersion, as shown by averaged regression coefficients between 1.005 and 1.032 (Figs. 3 and 6). Finally, the PA approach was the least accurate and showed the highest dispersion and level biases (Figs. 2, 3, 4, 5, 6 and 7).

## Discussion

In this study, first, we presented a unified model for ssGTBLUP and ssSNPBLUP, and we introduced different approaches to predict GEBV of genotyped selection candidates based on solutions of a single-step evaluation that does not consider the pedigree and genotypes of these selection candidates indirectly. The performance of the different models and methods was investigated using a dataset with a total of 2.61 million genotypes. In this section, we will discuss the following three points: (1) the computational similarities and differences between ssGTBLUP and ssSNPBLUP; (2) the indirect prediction of GEBV; and (3) the performance gains by ignoring genotypes of selection candidates in the single-step evaluation models.

### Computational similarities and differences between ssGTBLUP and ssSNPBLUP

Within a PCG iteration, the computations needed in MME (2) and (3) are theoretically similar. The main computational task in each iteration of the PCG method is the MME coefficient matrix times a vector product. Any differences in the necessary MME coefficient matrix times a vector product in MME (2) and (3) are due to differences in ${\mathbf{H}}^{-1}$ and ${\mathbf{H}}_{{\varvec{L}}}^{-1}$. However, these products can be arranged so that they perform with similar efficiency, as shown with the component-wise approach of ssGTBLUP. The use of ${\mathbf{T}}_{a}$ in the current implementation of MiXBLUP explains the RAM and time differences observed between the original ssGTABLUP and the component-wise ssGTABLUP and ssSNPBLUP in MiXBLUP. As expected, the component-wise ssGTABLUP approach and ssSNPBLUP require similar times per PCG iteration as well as similar RAM. The differences in RAM between these two approaches can be explained by the storage of $\mathbf{L}$ in RAM as a double precision dense matrix that requires $8{m}^{2}$ bytes. Consequently, our results illustrate that the component-wise ssGTBLUP (MME (2)) approach and ssSNPBLUP (MME (3)) result in performing a similar number of floating-point operations.

Vandenplas et al. [26] showed that the multiplication of $\mathbf{Z}$ by an array with the use of $\mathbf{M}$ stored using the Plink 1 binary form was more than twice as fast compared to the same multiplication using the Intel MKL DGEMM subroutine, thanks to the efficient use of parallelization, vectorization, and CPU cache with the packed $\mathbf{M}$ matrix. The efficiency of the packed matrix operations is also indicated by the fact that ssGTABLUP and ssSNPBLUP approaches showed a similar reduction in computing time per iteration in the solver step when analysing the reduced data instead of the full data. This illustrates the fact that both approaches behave numerically similarly when the number of genotyped animals changes.

Although the computations needed for ssGTABLUP and ssSNPBLUP within a PCG iteration are theoretically similar, both ssGTABLUP approaches needed a longer total computing time than for ssSNPBLUP. This longer time is mainly due to the heavy preprocessing step to compute ${\mathbf{T}}_{a}$ for the original ssGTABLUP approach and $\mathbf{L}$ for the component-wise ssGTABLUP approach. Both matrices are computed with the same procedure of the program calc_grm, except that the last steps for computing ${\mathbf{T}}_{a}$ from $\mathbf{L}$ and writing it to a file, are skipped for the component-wise ssGTABLUP approach. This current implementation explains the reduced times and similar amounts of RAM for the preprocessing step of the component-wise ssGTABLUP approach in comparison to the original ssGTABLUP approach, as both computations use a genotype matrix stored in a 8-byte array. These preprocessing costs could be reduced by computing a single ${\mathbf{T}}_{a}$ or $\mathbf{L}$ for multiple genomic evaluations that share a common set of genotyped animals. Since ssSNPBLUP has no such preprocessing step, for this model it is more attractive to define minimal sets of required genotypes for each genomic evaluation separately, by removing genotypes of animals that had neither own nor progeny phenotypes, as done here.

Finally, both ${\mathbf{H}}^{-1}$ and ${\mathbf{H}}_{{\varvec{L}}}^{-1}$ contain $\mathbf{K}$. Because the size of this matrix is limited by the number of markers $m$ when the SNP effects are assumed to have the same weights across all traits, this dense matrix can be stored in computer RAM. In MME (2), its inverse, ${\mathbf{K}}^{-1}$, or its Cholesky decomposition ${\mathbf{L}}_{C}$, can be precomputed to gain efficiency in the PCG iterations. It is worth noting that MME (3) can be more easily applicable for a model where $\mathbf{K}$ is not the same across the traits because $\mathbf{K}$ does not need to be inverted as in MME (2). Then, instead of precomputing $\mathbf{K}$ for each trait, a computationally more efficient approach for MME (3) can be the computation of the needed matrix times vector product during the PCG iteration. Consider the product $\mathbf{K}\mathbf{g}=\mathbf{Z}^{\mathbf{\prime}}{\mathbf{C}}^{-1}\mathbf{Z}\mathbf{g}+{\mathbf{B}}^{-1}\mathbf{g}$. Because the $\mathbf{s}=\mathbf{Z}\mathbf{g}$ product needs to be computed at each iteration, and because the number of iterations is typically much smaller than the number of SNPs, the multiplication of $\mathbf{Z}^{\mathbf{\prime}}\left({\mathbf{C}}^{-1}\mathbf{s}\right)$ during each iteration is less demanding than precomputing once the product $\mathbf{Z}^{\mathbf{\prime}}{\mathbf{C}}^{-1}\mathbf{Z}$ [26].

The ssGTABLUP approach showed a better convergence than the ssSNPBLUP approach. This can be attributed to a better preconditioner in ssGTABLUP. In the ssGTABLUP, the diagonal of the MME matrix is easier to compute than in the ssSNPBLUP approach where the diagonal for the marker effects is approximated [26], because $\mathbf{K}$ is not computed explicitly as done for ssGTABLUP. The computation of $\mathbf{K}$ for ssSNPBLUP would lead to a similar preprocessing time as for ssGTABLUP and could allow faster convergence. However, the total computing time for ssSNPBLUP would be increased. This illustrates that the preconditioner is a compromise that is achieved within the tolerated preprocessing computing time.

### Indirect prediction of GEBV

As shown by our results, the GRV and REG approaches proposed in this study for predicting indirectly and efficiently GEBV of genotyped selection candidates are accurate and (almost) unbiased. The accuracy and unbiasedness of the GRV and REG approaches can be explained by the fact that all components of a GEBV are adequately computed by the proposed indirect prediction approaches. Approximating some components of the GEBV, such as the RPG and SNP effects, or ignoring some of them, such as the contribution of the J-factor, may result in less accurate and more biased indirect GEBV. Hereinafter we discuss alternatives illustrated by the indirect approaches that approximate the GEBV.

The SNP effects can be computed from GEBV estimated with ssGTBLUP without any approximations using Eq. (5), that is $\widehat{\mathbf{g}}={\mathbf{K}}^{-1}\mathbf{Z}^{\mathbf{\prime}}{\mathbf{C}}^{-1}{\widehat{\mathbf{u}}}_{g}$. This Eq. (5) yields exact solutions for $\widehat{\mathbf{g}}$ and can be efficiently implemented for all versions of ssGBLUP, in contrast to the equation $\widehat{\mathbf{g}}=\mathbf{B}\mathbf{Z}^{\mathbf{\prime}}{\mathbf{G}}_{C}^{-1}{\widehat{\mathbf{u}}}_{g}$ requiring ${\mathbf{G}}_{C}^{-1}$ and often used in the literature [12, 16, 23]. While it can be shown that the equation $\widehat{\mathbf{g}}=\mathbf{B}\mathbf{Z}\mathrm{^{\prime}}{\mathbf{G}}_{C}^{-1}{\widehat{\mathbf{u}}}_{g}$ is equivalent to Eq. (5), its implementation for large genotyped datasets requires an approximated ${\mathbf{G}}_{C}^{-1}$ [16, 23], resulting in an approximated $\widehat{\mathbf{g}}$. An alternative approach to compute $\widehat{\mathbf{g}}$ without computing ${\mathbf{G}}_{C}^{-1}$ has been proposed by Pimentel et al. [12]. This approach consists of solving a SNPBLUP model with GEBV as phenotypes. However, in the presence of an RPG effect, this approach still leads to an approximated $\widehat{\mathbf{g}}$ because it assumes implicitly that $\mathbf{C}=\upvarepsilon \mathbf{I}$ instead of $\mathbf{C}=w{\mathbf{A}}_{gg}$.

The computation of the RPG effects for the selection candidates can be efficiently implemented with our exact alternative approach to Eq. (6), or with the approximated REG approach that uses a mean parent RPG value when the genotyped parent’s RPG value is known but a regression approach is used for a non-genotyped parent. Ignoring the RPG effects leads to dispersion bias of the indirect GEBV, as shown by the results for the DGV approach. The proposed exact approach gains efficiency because it requires neither ${\mathbf{G}}_{C}^{-1}$ [16, 23] nor ${\mathbf{A}}_{gg}^{-1}$ [12, 13], as previously proposed in the literature.

Although our REG approach resulted in accurate indirect GEBV for the selection candidates, other approaches for approximating the RPG effects for the selection candidates have been proposed or could be tested. For example, Lourenco et al. [23] proposed to use the linear regression of individual GEBV on direct genomic values, i.e., estimate $a$ and $b$ in the linear regression ${\widehat{\mathbf{u}}}_{g}=\widehat{a}+\widehat{b}\mathbf{Z}\widehat{\mathbf{g}}$, to compute directly GEBV of selection candidates as ${\widehat{\mathbf{u}}}_{c}=\widehat{a}+\widehat{b}{\mathbf{Z}}_{c}\widehat{\mathbf{g}}$. An alternative linear regression to that proposed in the "Methods" section here, would be to use the linear regression of individual on parent average GEBV, i.e., estimate $a$ and $b$ in the linear regression ${\widehat{\mathbf{d}}}_{g}=\widehat{a}+\widehat{b}{\widehat{\mathbf{u}}}_{g,PA}$ using genotyped animal values for ${\widehat{\mathbf{d}}}_{g}$ and ${\widehat{\mathbf{u}}}_{g,PA}$. Thus, the estimated coefficients $\widehat{a}$ and $\widehat{b}$ are used in the prediction equation for the candidate animals to estimate the RPG effects ${\widehat{\mathbf{d}}}_{c}=\widehat{a}+\widehat{b}{\widehat{\mathbf{u}}}_{c,PA}$ where the ${\widehat{\mathbf{u}}}_{c,PA}$ vector has parent average GEBV for the genotyped selection candidates. This approach has the advantage that the mean parent GEBV is used directly without having to know the genotyping status of either of the parents. Another alternative is to compute the RPG effect of the selection candidates as the three sire parent averages when the dam is missing [12]. Finally, it is worth noting that we used an RPG proportion of 0.20 in this study. The use of an RPG proportion close to 0 will improve the accuracy and the dispersion bias of the indirect GEBV by decreasing the importance of ${\widehat{\mathbf{d}}}_{c}$ and increasing the importance of DGV.

No level bias was observed with the GRV method, and the bias was negligible for the REG approach. In the presence of level bias, animals with indirect GEBV cannot be compared to animals with GEBV computed by a single-step evaluation. Level bias can be attributed to unaccounted differences between the pedigree and genomic bases [12, 16, 23], as well as to approximated RPG effects (but with a smaller contribution than the first one), as illustrated by the DGV and REG approaches. The issue of level bias was solved in the literature with different approaches, such as by adding the mean GEBV of the genotyped animals of the previous single-step evaluation [23], or by adding a general mean that is estimated simultaneously with the estimates of SNP effects using GEBV of genotyped animals [12]. The mean computed by these two approaches can be considered as an approximation of the J-factor covariate fitted explicitly in this study. For ssGBLUP approaches in which the J-factor effect is fitted as a random effect and absorbed in the additive genetic effect [20], its value can be easily computed from the GEBV of the genotyped animals as:

$$\widehat{\mu }=k{\mathbf{1}}^{\mathbf{\prime}}{\mathbf{G}}_{C}^{-1}{\widehat{\mathbf{u}}}_{g}=k{\mathbf{1}}^{\mathbf{\prime}}\left({\mathbf{C}}^{-1}-{\mathbf{C}}^{-1}\mathbf{Z}{\mathbf{K}}^{-1}{\mathbf{Z}}^{\mathbf{\prime}}{\mathbf{C}}^{-1}\right){\widehat{\mathbf{u}}}_{g}$$

$$=k{\mathbf{1}}^{\mathbf{\prime}}{\mathbf{C}}^{-1}\left({\widehat{\mathbf{u}}}_{g}-\mathbf{Z}\widehat{\mathbf{g}}\right)=k{\mathbf{1}}^{\mathbf{\prime}}{\mathbf{C}}^{-1}{\widehat{\mathbf{d}}}_{g},$$

where $k$ is a function of the proportion of RPG and the variance of the J-factor effect [20].

### Performance gains by ignoring the genotypes of selection candidates

Ignoring the genotypes of the selection candidates, that is genotyped animals without own or progeny records, can be an effective method to reduce the computational costs of the single-step genomic evaluations in some single-step genomic evaluations. In our study, ignoring 17% of the whole genotype set decreased the computing time for ${\mathbf{T}}_{a}$, and the computing time per iteration of ssGTABLUP and ssSNPBLUP by between 17 and 23%, while leading to almost the same GEBV for the animals included in both the reduced and full evaluations. The observed reductions in computing time per iteration matches our expectations. The main computational costs within each iteration are due to the multiplication of ${\mathbf{T}}_{a}$ (or $\mathbf{Z}$) and its transpose by an array, for which the computational costs depend linearly on the number of genotyped animals. Therefore, it will be easy to estimate the potential performance gains by ignoring the genotypes of selection candidates in routine single-step genomic evaluations.

It is worth noting that small GEBV differences can be expected between the reduced and the full single-step evaluations for non-genotyped parents of selection candidates, as the genotypes of their non-included progeny will not contribute to the imputation of their genotype within the single-step evaluations. The impact of this effect could be larger and should be further investigated, e.g., for species with large litters because the accuracy of the imputed genotype of a non-genotyped parent increases with the number of genotyped offspring [32].

## Conclusions

In this study, first, we presented a unified model for two single-step approaches, ssGTBLUP and ssSNPBLUP. Second, we presented different approaches to predict indirectly GEBV of genotyped selection candidates based on solutions of a single-step evaluation that does not consider the genotypes of these selection candidates. Based on our results, ignoring genotypes of selection candidates resulted in faster single-step evaluations, and the proposed indirect approaches resulted in accurate indirect GEBV for selection candidates, with almost no dispersion and level bias. The proposed indirect approaches are also more memory efficient and computationally fast, compared to solving the single-step evaluations. Therefore, they can be computed even on a weekly basis to estimate GEBV for newly genotyped animals while the full single-step evaluation is done only a few times within a year.

## Supplementary Information

**Additional file 1: Table S1.** Pearson correlations, regression coefficients, and level bias for direct and maternal GEBV computed from the full ssSNPBLUP versus from the indirect prediction approaches for 251,332 genotyped selection candidates with both parents genotyped. **Table S2.** Pearson correlations, regression coefficients, and level bias for direct and maternal GEBV computed from the full ssSNPBLUP versus from the indirect prediction approaches for 155,675 genotyped selection candidates with only one genotyped parent. **Table S3.** Pearson correlations, regression coefficients, and level bias for direct and maternal GEBV computed from the full ssSNPBLUP versus from the indirect prediction approaches for 50,164 genotyped selection candidates with no genotyped parents. **Table S4.** Pearson correlations, regression coefficients, and level bias for direct and maternal GEBV computed from the full ssGTABLUP versus from the indirect prediction approaches for 251,332 genotyped selection candidates with both parents genotyped. **Table S5.** Pearson correlations, regression coefficients, and level bias for direct and maternal GEBV computed from the full ssGTABLUP versus from the indirect prediction approaches for 155,675 genotyped selection candidates with only one genotyped parent. **Table S6.** Pearson correlations, regression coefficients, and level bias for direct and maternal GEBV computed from the full ssGTABLUP versus from the indirect prediction approaches for 50,164 genotyped selection candidates with no genotyped parents.
