# Supplementary material for: Efficient large-scale single-step evaluations and indirect genomic prediction of genotyped selection candidates
Source: Genet Sel Evol. 2023 Jun 8;55:37. doi: 10.1186/s12711-023-00808-z (PMC10251624; doi:10.1186/s12711-023-00808-z)
Supplement: Supplementary file 1 — Additional file 1: Table S1. Pearson correlations, regression coefficients, and level bias for direct and maternal GEBV computed from the full ssSNPBLUP versus from the indirect prediction approaches for 251,332 genotyped selection candidates with both parents genotyped. Table S2. Pearson correlations, regression coefficients, and level bias for direct and maternal GEBV computed from the full ssSNPBLUP versus from the indirect prediction approaches for 155,675 genotyped selection candidates with only one genotyped parent. Table S3. Pearson correlations, regression coefficients, and level bias for direct and maternal GEBV computed from the full ssSNPBLUP versus from the indirect prediction approaches for 50,164 genotyped selection candidates with no genotyped parents. Table S4. Pearson correlations, regression coefficients, and level bias for direct and maternal GEBV computed from the full ssGTABLUP versus from the indirect prediction approaches for 251,332 genotyped selection candidates with both parents genotyped. Table S5. Pearson correlations, regression coefficients, and level bias for direct and maternal GEBV computed from the full ssGTABLUP versus from the indirect prediction approaches for 155,675 genotyped selection candidates with only one genotyped parent. Table S6. Pearson correlations, regression coefficients, and level bias for direct and maternal GEBV computed from the full ssGTABLUP versus from the indirect prediction approaches for 50,164 genotyped selection candidates with no genotyped parents. [file 12711_2023_808_MOESM1_ESM.pdf]

Additional file 1

Table S1. Pearson correlations, regression coefficients, and level bias for direct and maternal GEBV computed from the full ssSNPBLUP versus from the indirect prediction approaches for 251,332 genotyped selection candidates with both parents genotyped.

Effect	Parameter	Approach[1]	Trait						Average	SD
			1	2	3	4	5	6		
Direct	Correlation	PA	0.776	0.805	0.767	0.752	0.761	0.768	0.771	0.018
		DGV	0.955	0.965	0.940	0.930	0.929	0.969	0.948	0.017
		Regression	0.998	0.998	0.999	0.999	1.000	0.999	0.999	0.001
		GRV	0.998	0.998	0.999	0.999	1.000	0.999	0.999	0.001
	Reg. coeff.	PA	0.983	0.977	0.971	0.968	0.985	0.969	0.975	0.007
		DGV	1.027	1.043	1.033	1.027	1.020	1.024	1.029	0.008
		Regression	0.998	0.995	0.998	1.000	0.999	0.995	0.998	0.002
		GRV	0.998	0.995	0.998	1.000	0.999	0.995	0.998	0.002
	Level bias	PA	0.056	0.070	0.076	0.062	0.015	0.071	0.058	0.022
		DGV	0.010	0.160	0.159	0.214	-0.051	-0.143	0.058	0.141
		Regression	0.002	-0.001	0.001	0.000	0.003	-0.001	0.001	0.002
		GRV	0.002	-0.001	0.001	0.000	0.003	-0.001	0.001	0.002
Maternal	Correlation	PA	0.763	0.777	0.758	0.755	0.793	0.792	0.773	0.017
		DGV	0.977	0.970	0.964	0.957	0.963	0.977	0.968	0.008
		Regression	0.994	0.995	0.999	0.999	0.999	0.998	0.997	0.002
		GRV	0.994	0.995	0.999	0.999	0.999	0.998	0.997	0.002
	Reg. coeff.	PA	0.972	0.982	0.986	0.985	0.998	0.975	0.983	0.009
		DGV	1.026	1.041	1.034	1.034	1.039	1.018	1.032	0.008
		Regression	0.987	0.988	0.998	0.999	0.999	0.987	0.993	0.006
		GRV	0.987	0.988	0.998	0.999	0.999	0.987	0.993	0.006
	Level bias	PA	0.041	0.050	0.010	0.011	0.024	0.033	0.028	0.016
		DGV	0.239	0.101	0.213	0.184	0.091	0.200	0.171	0.061
		Regression	-0.010	0.005	0.012	0.010	-0.008	-0.008	0.000	0.010
		GRV	-0.010	0.005	0.012	0.010	-0.008	-0.008	0.000	0.010

[1] PA: parent average GEBVs; DGV: Direct genomic values; Regression: GEBVs with approximated residual polygenic effects; and GRV: exact computation of GEBVs.

Table S2. Pearson correlations, regression coefficients, and level bias for direct and maternal GEBV computed from the full ssSNPBLUP versus from the indirect prediction approaches for 155,675 genotyped selection candidates with only one genotyped parent.

Effect	Parameter	Approach[1]	Trait						Average	SD
			1	2	3	4	5	6		
Direct	Correlation	PA	0.674	0.713	0.682	0.674	0.680	0.683	0.684	0.015
		DGV	0.950	0.957	0.938	0.936	0.935	0.971	0.948	0.015
		Regression	0.990	0.991	0.991	0.992	0.992	0.994	0.992	0.001
		GRV	0.998	0.998	0.998	0.999	0.999	0.998	0.998	0.000
	Reg. coeff.	PA	0.987	0.970	0.960	0.950	0.964	0.988	0.970	0.015
		DGV	1.004	1.024	1.032	1.038	1.034	1.025	1.026	0.012
		Regression	0.994	0.996	1.000	0.998	0.997	0.993	0.997	0.003
		GRV	1.000	0.996	1.000	1.000	0.998	0.994	0.998	0.003
	Level bias	PA	0.080	0.135	0.100	0.083	0.007	0.022	0.071	0.048
		DGV	-0.026	0.121	0.127	0.190	-0.083	-0.159	0.029	0.138
		Regression	-0.009	-0.013	-0.001	0.006	-0.004	-0.003	-0.004	0.006
		GRV	0.005	-0.001	0.003	0.002	0.006	-0.001	0.002	0.003
Maternal	Correlation	PA	0.639	0.669	0.639	0.653	0.665	0.654	0.653	0.013
		DGV	0.972	0.967	0.965	0.959	0.950	0.968	0.963	0.008
		Regression	0.988	0.990	0.994	0.993	0.989	0.990	0.991	0.002
		GRV	0.994	0.995	0.998	0.998	0.998	0.997	0.997	0.002
	Reg. coeff.	PA	0.932	0.964	0.914	0.937	0.991	0.958	0.949	0.027
		DGV	1.023	1.043	1.037	1.044	0.978	0.991	1.019	0.028
		Regression	0.995	0.999	1.003	1.006	0.981	0.984	0.995	0.010
		GRV	0.993	0.990	0.998	1.001	0.999	0.991	0.995	0.005
	Level bias	PA	0.083	0.051	0.083	0.070	0.020	0.058	0.061	0.024
		DGV	0.263	0.126	0.232	0.197	0.115	0.239	0.195	0.062
		Regression	0.010	0.023	0.024	0.021	0.012	0.017	0.018	0.006
		GRV	-0.003	0.009	0.012	0.010	-0.004	-0.001	0.004	0.007

[1] PA: parent average GEBVs; DGV: Direct genomic values; Regression: GEBVs with approximated residual polygenic effects; and GRV: exact computation of GEBVs.

Table S3. Pearson correlations, regression coefficients, and level bias for direct and maternal GEBV computed from the full ssSNPBLUP versus from the indirect prediction approaches for 50,164 genotyped selection candidates with no genotyped parents.

Effect	Parameter	Approach[1]	Trait						Average	SD
			1	2	3	4	5	6		
Direct	Correlation	PA	0.420	0.437	0.439	0.461	0.454	0.443	0.442	0.014
		DGV	0.968	0.969	0.964	0.970	0.970	0.986	0.971	0.008
		Regression	0.975	0.977	0.976	0.981	0.980	0.988	0.980	0.005
		GRV	0.996	0.996	0.996	0.997	0.997	0.997	0.996	0.001
	Reg. coeff.	PA	0.860	0.840	0.799	0.780	0.811	0.885	0.829	0.039
		DGV	0.998	1.011	1.010	1.012	1.008	0.995	1.005	0.007
		Regression	0.986	0.996	0.987	0.975	0.978	0.978	0.983	0.008
		GRV	1.001	0.998	1.003	1.002	0.999	0.993	0.999	0.003
	Level bias	PA	0.050	0.141	0.103	0.105	-0.037	-0.039	0.054	0.077
		DGV	-0.035	0.117	0.112	0.167	-0.113	-0.173	0.012	0.139
		Regression	-0.019	-0.022	-0.007	0.001	-0.018	-0.018	-0.014	0.009
		GRV	0.008	0.000	0.005	0.004	0.007	-0.003	0.003	0.004
Maternal	Correlation	PA	0.367	0.453	0.356	0.393	0.369	0.357	0.383	0.037
		DGV	0.982	0.980	0.982	0.978	0.972	0.984	0.980	0.004
		Regression	0.985	0.985	0.987	0.986	0.976	0.986	0.984	0.004
		GRV	0.992	0.994	0.997	0.997	0.998	0.997	0.996	0.002
	Reg. coeff.	PA	0.744	0.884	0.654	0.735	0.837	0.771	0.771	0.081
		DGV	1.014	1.024	1.019	1.024	0.983	0.995	1.010	0.017
		Regression	1.004	1.006	1.004	1.007	0.973	0.985	0.996	0.014
		GRV	0.998	0.994	0.998	1.001	0.999	0.994	0.997	0.003
	Level bias	PA	0.120	0.032	0.144	0.109	0.029	0.108	0.090	0.048
		DGV	0.251	0.110	0.236	0.196	0.088	0.222	0.184	0.068
		Regression	0.008	0.023	0.027	0.024	0.007	0.013	0.017	0.009
		GRV	-0.001	0.012	0.010	0.010	-0.004	-0.001	0.004	0.007

[1] PA: parent average GEBVs; DGV: Direct genomic values; Regression: GEBVs with approximated residual polygenic effects; and GRV: exact computation of GEBVs.

Table S4. Pearson correlations, regression coefficients, and level bias for direct and maternal GEBV computed from the full ssGTABLUP versus from the indirect prediction approaches for 251,332 genotyped selection candidates with both parents genotyped.

Effect	Parameter	Approach[1]	Trait						Average	SD
			1	2	3	4	5	6		
Direct	Correlation	PA	0.776	0.804	0.767	0.754	0.761	0.764	0.771	0.018
		DGV	0.956	0.965	0.940	0.929	0.930	0.965	0.947	0.017
		Regression	0.998	0.998	0.999	0.999	0.999	0.996	0.998	0.001
		GRV	0.998	0.998	0.999	0.999	0.999	0.996	0.998	0.001
	Reg. coeff.	PA	0.983	0.974	0.972	0.973	0.986	0.967	0.976	0.007
		DGV	1.026	1.040	1.034	1.029	1.022	1.024	1.029	0.007
		Regression	0.998	0.994	0.999	1.002	1.000	0.994	0.998	0.003
		GRV	0.998	0.994	0.999	1.002	1.000	0.994	0.998	0.003
	Level bias	PA	0.061	0.077	0.077	0.064	0.016	0.073	0.061	0.023
		DGV	0.010	0.160	0.160	0.216	-0.049	-0.136	0.060	0.140
		Regression	0.007	0.005	0.002	0.002	0.004	0.001	0.003	0.002
		GRV	0.007	0.005	0.002	0.002	0.004	0.001	0.003	0.002
Maternal	Correlation	PA	0.758	0.771	0.754	0.754	0.793	0.784	0.769	0.016
		DGV	0.975	0.969	0.962	0.956	0.962	0.973	0.966	0.008
		Regression	0.990	0.993	0.997	0.998	0.998	0.994	0.995	0.003
		GRV	0.990	0.993	0.997	0.998	0.998	0.994	0.995	0.003
	Reg. coeff.	PA	0.951	0.974	0.980	0.988	0.990	0.947	0.972	0.018
		DGV	1.013	1.031	1.028	1.033	1.034	0.999	1.023	0.014
		Regression	0.974	0.982	0.994	1.001	0.994	0.969	0.986	0.013
		GRV	0.974	0.982	0.994	1.001	0.994	0.969	0.986	0.013
	Level bias	PA	0.034	0.050	0.016	0.012	0.023	0.031	0.028	0.014
		DGV	0.220	0.110	0.214	0.183	0.088	0.189	0.167	0.055
		Regression	-0.017	0.004	0.017	0.011	-0.009	-0.011	-0.001	0.013
		GRV	-0.017	0.004	0.017	0.011	-0.009	-0.011	-0.001	0.013

[1] PA: parent average GEBVs; DGV: Direct genomic values; Regression: GEBVs with approximated residual polygenic effects; and GRV: exact computation of GEBVs.

Table S5. Pearson correlations, regression coefficients, and level bias for direct and maternal GEBV computed from the full ssGTABLUP versus from the indirect prediction approaches for 155,675 genotyped selection candidates with only one genotyped parent.

Effect	Parameter	Approach[1]	Trait						Average	SD
			1	2	3	4	5	6		
Direct	Correlation	PA	0.676	0.713	0.686	0.679	0.680	0.678	0.685	0.014
		DGV	0.949	0.957	0.938	0.934	0.935	0.968	0.947	0.014
		Regression	0.990	0.991	0.991	0.991	0.992	0.992	0.991	0.001
		GRV	0.997	0.997	0.998	0.998	0.998	0.996	0.997	0.001
	Reg. coeff.	PA	0.989	0.965	0.968	0.960	0.968	0.979	0.971	0.011
		DGV	1.002	1.018	1.038	1.040	1.038	1.019	1.026	0.015
		Regression	0.994	0.992	1.005	1.001	1.000	0.987	0.996	0.007
		GRV	1.000	0.993	1.004	1.003	1.000	0.988	0.998	0.006
	Level bias	PA	0.085	0.137	0.104	0.089	0.012	0.027	0.076	0.048
		DGV	-0.024	0.121	0.132	0.200	-0.075	-0.148	0.034	0.137
		Regression	-0.005	-0.011	0.003	0.013	0.001	0.001	0.000	0.008
		GRV	0.008	0.000	0.006	0.009	0.010	0.002	0.006	0.004
Maternal	Correlation	PA	0.639	0.664	0.634	0.654	0.665	0.648	0.650	0.013
		DGV	0.970	0.966	0.963	0.957	0.943	0.963	0.960	0.010
		Regression	0.986	0.989	0.991	0.991	0.984	0.987	0.988	0.003
		GRV	0.992	0.994	0.996	0.997	0.997	0.994	0.995	0.002
	Reg. coeff.	PA	0.923	0.956	0.916	0.947	0.982	0.934	0.943	0.024
		DGV	1.019	1.035	1.034	1.045	0.968	0.975	1.013	0.033
		Regression	0.989	0.994	1.001	1.008	0.974	0.969	0.989	0.015
		GRV	0.987	0.987	0.999	1.005	0.994	0.977	0.991	0.010
	Level bias	PA	0.074	0.055	0.090	0.075	0.024	0.056	0.062	0.023
		DGV	0.248	0.137	0.231	0.197	0.117	0.231	0.193	0.054
		Regression	0.005	0.024	0.028	0.024	0.018	0.016	0.019	0.008
		GRV	-0.008	0.010	0.018	0.015	0.002	-0.003	0.006	0.010

[1] PA: parent average GEBVs; DGV: Direct genomic values; Regression: GEBVs with approximated residual polygenic effects; and GRV: exact computation of GEBVs.

Table S6. Pearson correlations, regression coefficients, and level bias for direct and maternal GEBV computed from the full ssGTABLUP versus from the indirect prediction approaches for 50,164 genotyped selection candidates with no genotyped parents.

Effect	Parameter	Approach[1]	Trait						Average	SD
			1	2	3	4	5	6		
Direct	Correlation	PA	0.424	0.435	0.447	0.470	0.449	0.435	0.443	0.016
		DGV	0.967	0.968	0.963	0.969	0.970	0.983	0.970	0.007
		Regression	0.975	0.976	0.976	0.980	0.981	0.985	0.979	0.004
		GRV	0.995	0.995	0.995	0.996	0.997	0.995	0.996	0.001
	Reg. coeff.	PA	0.865	0.837	0.810	0.793	0.806	0.856	0.828	0.029
		DGV	0.997	1.009	1.013	1.012	1.008	0.983	1.004	0.012
		Regression	0.985	0.995	0.990	0.975	0.979	0.965	0.981	0.011
		GRV	1.001	0.996	1.004	1.003	0.999	0.982	0.997	0.008
	Level bias	PA	0.054	0.142	0.105	0.108	-0.034	-0.043	0.055	0.078
		DGV	-0.032	0.119	0.116	0.173	-0.108	-0.168	0.017	0.139
		Regression	-0.017	-0.021	-0.005	0.006	-0.015	-0.020	-0.012	0.010
		GRV	0.008	-0.003	0.005	0.008	0.009	-0.006	0.004	0.006
Maternal	Correlation	PA	0.372	0.446	0.345	0.393	0.375	0.348	0.380	0.037
		DGV	0.980	0.979	0.981	0.977	0.967	0.981	0.977	0.005
		Regression	0.983	0.984	0.986	0.984	0.971	0.982	0.982	0.005
		GRV	0.991	0.993	0.996	0.996	0.997	0.994	0.995	0.002
	Reg. coeff.	PA	0.755	0.874	0.647	0.742	0.841	0.739	0.766	0.081
		DGV	1.015	1.020	1.016	1.024	0.977	0.980	1.005	0.021
		Regression	1.005	1.003	1.001	1.006	0.968	0.970	0.992	0.018
		GRV	0.998	0.991	0.998	1.003	0.997	0.980	0.995	0.008
	Level bias	PA	0.112	0.046	0.147	0.113	0.029	0.102	0.092	0.045
		DGV	0.240	0.126	0.236	0.198	0.087	0.212	0.183	0.063
		Regression	0.007	0.027	0.028	0.027	0.009	0.009	0.018	0.011
		GRV	-0.004	0.015	0.011	0.012	-0.003	-0.006	0.004	0.009

[1] PA: parent average GEBVs; DGV: Direct genomic values; Regression: GEBVs with approximated residual polygenic effects; and GRV: exact computation of GEBVs.
